# A New Chronology for Rhafas, Northeast Morocco, Spanning the North African Middle Stone Age through to the Neolithic

**DOI:** 10.1371/journal.pone.0162280

**Published:** 2016-09-21

**Authors:** Nina Doerschner, Kathryn E. Fitzsimmons, Peter Ditchfield, Sue J. McLaren, Teresa E. Steele, Christoph Zielhofer, Shannon P. McPherron, Abdeljalil Bouzouggar, Jean-Jacques Hublin

**Affiliations:** 1 Department of Human Evolution, Max Planck Institute for Evolutionary Anthropology, Leipzig, Germany; 2 Research Laboratory for Archaeology and the History of Art, University of Oxford, Oxford, United Kingdom; 3 Department of Geography, University of Leicester, Leicester, United Kingdom; 4 Department of Anthropology, University of California Davis, Davis, California, United States of America; 5 Institute of Geography, University of Leipzig, Leipzig, Germany; 6 Institut National des Sciences de l'Archéologie et du Patrimoine, Rabat, Morocco; 7 Institute of Advanced Study, Aix-Marseille University, Marseille, France; Universidade do Algarve, PORTUGAL

## Abstract

Archaeological sites in northern Africa provide a rich record of increasing importance for the origins of modern human behaviour and for understanding human dispersal out of Africa. However, the timing and nature of Palaeolithic human behaviour and dispersal across north-western Africa (the Maghreb), and their relationship to local environmental conditions, remain poorly understood. The cave of Rhafas (northeast Morocco) provides valuable chronological information about cultural changes in the Maghreb during the Palaeolithic due to its long stratified archaeological sequence comprising Middle Stone Age (MSA), Later Stone Age (LSA) and Neolithic occupation layers. In this study, we apply optically stimulated luminescence (OSL) dating on sand-sized quartz grains to the cave deposits of Rhafas, as well as to a recently excavated section on the terrace in front of the cave entrance. We hereby provide a revised chronostratigraphy for the archaeological sequence at the site. We combine these results with geological and sedimentological multi-proxy investigations to gain insights into site formation processes and the palaeoenvironmental record of the region. The older sedimentological units at Rhafas were deposited between 135 ka and 57 ka (MIS 6 –MIS 3) and are associated with the MSA technocomplex. Tanged pieces start to occur in the archaeological layers around 109 ka, which is consistent with previously published chronological data from the Maghreb. A well indurated duricrust indicates favourable climatic conditions for the pedogenic cementation by carbonates of sediment layers at the site after 57 ka. Overlying deposits attributed to the LSA technocomplex yield ages of ~21 ka and ~15 ka, corresponding to the last glacial period, and fall well within the previously established occupation phase in the Maghreb. The last occupation phase at Rhafas took place during the Neolithic and is dated to ~7.8 ka.

## Introduction

In recent years, data from cave sites in the Maghreb (comprising Morocco, Algeria, Tunisia and western Libya) have gain considerable importance in the study of modern human origins and dispersals within and out of Africa [1]. Not only are these sites relatively plentiful and their locations highly strategic, rich faunal and archaeological records are often well preserved within stratified sedimentological sequences (e.g. [2, 3]). This situation provides optimal conditions for the successful combination of classical archaeological methods with chronometric dating and palaeoenvironmental reconstruction. Despite this, there are few sites in this region that span multiple Palaeolithic technocomplexes.

Interest in the chronology of North African archaeological sites has arisen in part because of evidence for the early appearance of symbolic artefacts and other behavioural indicators–present in the Maghreb—interpreted to represent cultural modernity and which may be linked to the dispersal of anatomically modern humans from Africa [4–7]. The timing and geographic distribution of the emergence of these behaviours is of critical importance for modelling the drivers of population mobilisation and the eventual replacement of other human species [1].

In North Africa, particular interest is placed on an MSA technocomplex known as the Aterian (S1 File). Although the Aterian is primarily known for its pedunculated tools and bifacial foliates, that Aterian assemblages can additionally be characterised by the presence of blades, bladelets, end-scrapers, small Levallois cores [8] and the appearance of shell beads and other personal ornaments [5]. However, while the definition and concept of the Aterian is better defined today, there are still issues that remain, especially in northwest Africa [8, 9]. While current definitions recognize that there is more to the Aterian than tanged pieces, there remains the difficulty of reliably distinguishing the Aterian from the North African MSA when these characteristic finds are absent (e.g. [1, 10]). When the chronological position of the Aterian was thought to fall within an age range of 40–20 thousands of years ago (ka) [11], meaning clearly post non-Aterian MSA, its status as a distinct entity seemed clearer. However, more recent dating studies have extended the beginning of the Aterian to >100 ka [10, 12–17] and perhaps as early as 145±9 ka [10], which juxtaposes with the timing of the MSA in north Africa. Thus debate continues as to whether once the Aterian first occurs all subsequent assemblages are Aterian (meaning that the Aterian represents a phase within the MSA) or whether there is still a continuation of a non-Aterian MSA (meaning that the Aterian is a separate entity in North Africa) [9, 18].

The origins of the LSA are also of importance in North Africa; they are connected to a major change in human subsistence behaviour, as well as the emergence of elaborate funerary activities between ~40–20 ka [19, 20]. The LSA is characterised by the occurrence of microlithic bladelet industries, including large bladelets in the earliest phase labelled as Iberomaurusian in the Maghreb and “*Eastern Oranian*” in Libyan Cyrenaica [19, 21]. The LSA in the Maghreb starts ~22 kcal BP if not earlier [2], while it already appears >42 kcal BP in some sites [22, 23] elsewhere on the continent, but is–especially in South Africa—also a matter of ongoing debate (see e.g. [24]). Despite the substantial progress made in the last years [10, 16, 17, 25], there is clearly a need for additional data on the timing of the earliest LSA in the Maghreb.

**Fig 1:**
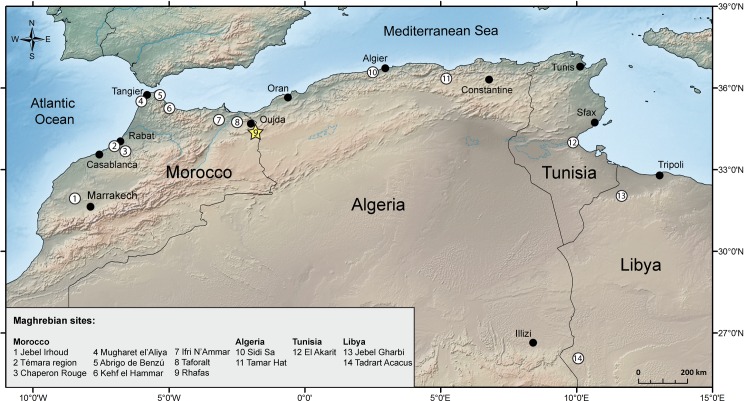
Map of archaeological sites cited in the text. The Témara region (2) includes the neighboring sites of El Mnasra, El Harhoura 1 & 2, Dar es-Soltan 1 & 2 and La Grotte de Contrebandiers; Jebel Gharbi (13) includes the sites of Ain Zargha, Jado, Shakshuk, Wadi Basina, Wadi Ghan and Wadi Sel; Tadrart Acacus (14) includes the sites of Uan Tabu and Uan Afuda.

The cave of Rhafas, located in north-eastern Morocco (Fig 1), is one of the few sites known to contain evidence of human occupation spanning the MSA, including the Aterian, through to the Neolithic. The first chronology for the upper layers of the cave fill sequence was produced by Mercier et al. [26] using both radiocarbon and luminescence dating techniques. ^14^C-dating gave ages of 5,963±150 cal BP (5,190±100 a BP, Gif-6185) for the uppermost Layer 1 (Neolithic) and 17,319±258 cal BP (14,060±150 a BP, Gif-6489) for Layer 2, although the latter date was considered incompatible with the archaeological context (Aterian). Thermoluminescence (TL) age estimates were obtained on burnt lithics from the sublevels of the MSA Layer 3 (92–60 ka). Additionally, one sediment sample from Layer 6d (also MSA) was dated by optically stimulated luminescence (OSL) on multiple grain aliquots using the 40–50 μm silt fraction (107±12 ka).

In this study, as part of renewed excavations, we build on the work by Mercier et al. [26] by applying single-grain OSL dating of quartz, providing a higher resolution, more complete chronostratigraphy for Rhafas. The application of ^14^C, while more precise than luminescence dating, is limited to dating the last 50 ka and cannot assist with constraining the timing of the older sediment deposits associated with the early emergence of MSA assemblages. OSL dating provides a reliable estimate of the time elapsed since mineral grains, such as quartz, were last exposed to sunlight [27], and therefore can be used to calculate the depositional age of sediments [15]. Although the first OSL ages for a Moroccan site (Chaperon Rouge I) were published by Texier in 1988 [28], the reliability of these ages is limited by the methods available at the time, such as aliquot size and lack of sensitivity change correction in the dating protocols. Major technical improvements in recent years [29, 30] have made optical dating of quartz the optimal tool for determining the age of sedimentological sequences in archaeological sites across Morocco [5, 6, 13–17, 26, 31–34]. Single grain dating has the advantage of enabling the identification of incomplete signal resetting, beta dose rate inconsistencies or post-depositional mixing of sediments, all of which are common in cave deposits (e.g. [16, 35, 36–39]). Each of these factors may result in significant over- or underestimation of the real depositional age of the sediment layer when using multi-grain OSL dating. Consequently, we have applied single grain dating to the Rhafas sequences to provide a more reliable chronology by identifying and mitigating potential problems in the luminescence signal which otherwise may not have been identified.

Thus here we present a chronology for the long, stratified, archaeological sequence at Rhafas based on OSL dating of individual quartz grains to better understand the temporal dimension of changes in human behaviour documented in the cave. We combine our chronological framework with geological and sedimentological analyses of the site’s’ sediments, a pedogenic carbonate crust and local bedrock samples to examine the depositional history of the sediment layers in further detail and to discuss the implications of the results in the context of Quaternary palaeoenvironmental change in the region.

### Regional setting

**Fig 2:**
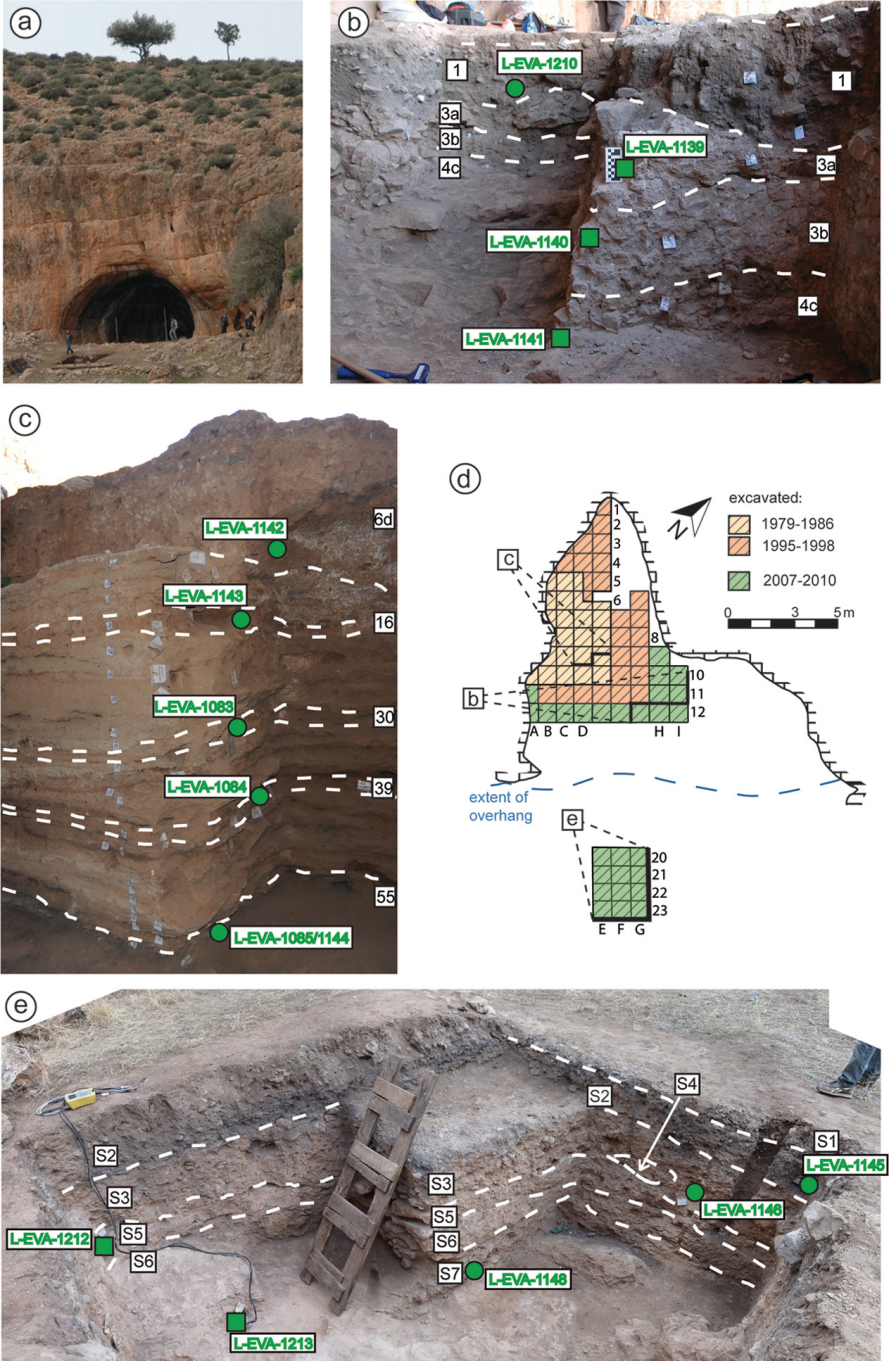
Overview of the Rhafas site and excavated sections. Photograph of the cave (a), plan view map with the excavation sectors (d) and photographs showing section walls of the cave mouth (b), the lower cave (c) and the terrace section (e). Indicated are layer boundaries and positions of the OSL samples.

Rhafas is a cave site ~900 m above present day sea level in the north-eastern Oujda Mountains, ~50 km inland from the Mediterranean coast and ~7 km west of the Algerian border (34°33'28''N, 1°52'26''W) (Fig 1). Situated on the north-western slope of a prominent northeast/southwest trending valley, the cave is a simple dome-shaped dissolution feature within the local dolomitic limestone, opening to the southeast. It has a maximum distance from the back wall to the current drip line of ~15 m, a width at the entrance of ~16 m, and a height of ~10 m (Fig 2).

#### Geological setting and cave formation

The local geology of the area is dominated by three main lithological units: a coarse-grained granodiorite intrusion forms the valley floor; a series of highly deformed low grade meta-sediments outcrop along the north-western slope of the valley; and an overlying dolomitic limestone sequence caps the local hilltops [40]. The meta-sediments are absent from the south-eastern side of the valley where the carbonate units rest directly on a weathered granodiorite surface. This may be related to the presence of a fault, with downthrow to the northwest running along the valley axis. The horizontally bedded dolomitic limestones at the north-western slope unconformably overlie the steeply dipping and strongly deformed meta-sediments and form a line of cliffs along both valley margins within which the cave is located. At the base of the cliff the unconformable contact is clearly exposed, and the outcrop of the unconformity can be traced below the cave entrance and further along the outcrop to the southwest.

The unconformity between the limestones and meta-sediments has acted as a locus for mineralisation within the area and is characterised by fractures infilled by carbonates, with abundant hematite mineralisation and localised lead sulphide precipitation. The unconformity is estimated to sit within 1–2 metres below the present excavated level of the cave floor. This has important implications for groundwater flow and dissolution of radiogenic isotopes from the granodiorite and metasediments into the lower parts of the cave fill which might have changed the radiation environment of these sediments subsequent to deposition. Consequently, it also has implications for environmental radiation dose rate determination for the lower cave sediments and, therefore, for the accuracy of the OSL ages.

The cave exhibits a simple morphology with no other entrance for sediments other than the current cave mouth and with only minor amounts of water reaching the cave from wall or roof seepage. However, on the eastern side of the cave mouth is a zone of fractured dolomitic limestone with closely spaced joints. This forms a prominent fabric (space cleavage) parallel with the main valley axis fault and possibly relating to late reactivation of this fault. This zone of fractured rock can be visually traced in front of the cave mouth where a largely buried boulder field forms the present hillslope. It is possible that this area of increased fracture porosity was the initial locus of dissolution and karstification with the present cave representing a lateral extension of this. If this was the case, then the dolomitic limestone boulders may represent the collapsed remains of an extension of the cave roof to the southeast. The timing of any such collapse remains unknown, but is likely to predate the deposition of sediments in the terrace area which appear to have vertically infilled the spaces between existing boulders.

#### History of archaeological research at Rhafas

The cave was first discovered in 1950 by J. Marion, and the stone tool assemblage was described by J. Roche [41] as similar to the “*Eastern Oranian*” or even earlier. The first series of systematic excavations were conducted from 1979 to 1986 by J.-L. Wengler [42, 43] over a large portion of the interior of the cave and to a depth of 4.5 m. Three additional seasons followed in the 1990s. In 2007, a new series of excavations forming the current campaign started in the cave as well as on the relatively flat and partially terraced area in front of the cave (Fig 2D and 2E) that separates the cave from the main valley slope.

The stratigraphy of the cave sediments was described by Wengler [42] on the basis of a reference section running from excavation square C4 to C10 (Fig 2D). 71 distinct sedimentary layers were identified, of which 39 are associated with archaeological finds. These were combined into four main units, numbered from top (I) to bottom (IV). Except for Unit I, all stratigraphic units end with important phases of carbonate formation (Layer 3, 6 and 69) and are separated by significant erosional breaks. The combined sequence within the cave contains artefact assemblages that can be attributed to the Neolithic (Layer 1) and the MSA, which was originally separated into Aterian (2, 3a) and Mousterian (3b to 71) Layers [42].

Although Wengler’s original section line no longer exists due to erosion and later excavation, his stratigraphic framework and numbering system have been retained. During the most recent excavations two additional reference profiles were developed to describe the cave mouth area and lower cave infill, respectively (Fig 2B and 2C). The current base of the cave does not reach Wengler’s Unit IV and ends at Layer 55 (Fig 2C). Additional units were opened and excavated on the level, approximately 25 m wide, terrace in front of the cave (terrace section, Fig 2E).

## Materials and Methods

Permission to undertake fieldwork and to collect bedrock and sediment samples at Rhafas was granted by the Institut National des Sciences de l'Archéologie et du Patrimoine, Rabat, Morocco.

### Luminescence dating. *OSL sampling and preparation*

Fifteen OSL samples were collected from three different sections at Rhafas, and span the entire archaeological sequence from MSA to Neolithic (Fig 2). Samples were either collected in stainless steel tubes (4 cm diameter, 10 cm long) or as blocks using hammer and chisel, depending on the degree of cementation of the layers. The samples were carefully sealed to preserve the field moisture content.

Gamma dose rate measurements were performed in situ with two portable sodium iodide gamma spectrometers and material surrounding the samples was collected for subsequent laboratory analysis. Gamma ray spectra were measured (except sample L-EVA-1139 from Layer 3a) using a three-inch crystal detector counting for 1800 s. Due to intensive cementation of the sediment layer, which made enlargement of the sampling hole difficult, a smaller, one-inch NaI detector was used for Layer 3a with a 5040 s counting interval.

Sample preparation and measurements were conducted in the luminescence laboratory of the Max Planck Institute for Evolutionary Anthropology under subdued red light conditions. The outer surfaces (~1 cm) of the block samples and 1.5 cm from both ends of the sampling tubes were removed because of potential light exposure during sampling. The remaining material was prepared to isolate pure sand-sized quartz grains for equivalent dose (D_e_) determination. After drying the block, samples were treated with hydrochloric acid (HCl, 10%) to dissolve carbonates. All samples were sieved to isolate the 90–212 μm in diameter sand fraction, which was used for further chemical treatments (removal of carbonates and organic matter with HCl (15%) and hydrogen peroxide (30%), respectively). Density separation was performed using a lithium heterotungstate solution (at 2.62 g cm^-3^ and 2.68 g cm^-3^ densities) in order to separate quartz from lighter feldspars and heavy minerals. The quartz was then treated with hydrofluoric acid (40%) for 60 min to etch the outer surface of the grains and to remove the remaining feldspar minerals. Finally, the samples were rinsed with HCl, dried and re-sieved to recover grains in size fractions of 90–125 μm, 125–180 μm and 180–212 µm in diameter.

#### Dose rate measurements

For the calculation of the external dose rate, high resolution germanium gamma spectrometry (HRGS), in situ gamma spectrometry and beta counting measurements were performed. The specific activities of radioactive elements ^238^U, ^232^Th, ^40^K and their daughter products (Table 1) were measured at the low-background underground laboratory Felsenkeller (VKTA, Dresden/Germany) using HRGS. Since this method measures the activities of multiple daughter isotopes within the uranium- and thorium-series decay chains, potential disequilibrium resulting from dissolution and transport of soluble daughter products–which causes time-dependent changes in the dose rate [44, 45]–can be identified. Comparisons of the ^238^U, ^226^Ra and ^210^Pb activities (S1 Fig) revealed no significant discrepancies indicating equilibrium for the uranium decay chain. It is, therefore, assumed that the dose rate of the sediments dated in this study remained constant through time. The conversion factors of Guérin et al. [46] were used to calculate the beta and gamma dose rates (S1 Table).

**Table 1:** Results of dose rate determination.

Sample	Depth	Moisture	Specific activities (Bq kg^-1^)					Dose rate (Gy/ka)				
	(cm)	content (%)	^238^U	^226^Ra	^210^Pb	^232^Th	^40^K	Beta^a^		Gamma^a^	Cosmic^a^	Total
Cave mouth section												
L-EVA-1210	40	10±5	15.7±1.9	14.5±0.7	13.5±1.6	10.5±0.5	207±11	0.90±0.02	0.34±0.02		0.05±0.01	1.29±0.07
L-EVA-1139	55	5±3	12.4±1.5	11.0±0.8	9.6±1.7	11.1±0.6	220±14	0.73±0.01	0.23±0.01		0.05±0.01	1.01±0.04
L-EVA-1140	70	5±3	9.6±1.4	8.7±0.8	8.8±2.9	6.4±0.4	105±9	0.69±0.03	0.22±0.01		0.05±0.01	0.96±0.04
L-EVA-1141	110	5±3	10.3±1.1	8.8±0.6	7.1±1.1	8.9±0.4	160±10	0.68±0.01	0.26±0.01		0.05±0.01	0.99±0.04
Lower cave section												
L-EVA-1142	185	5±3	15.4±3.2	15.7±1.1	12.9±2.7	18.8±1.0	931±53	1.49±0.02	0.61±0.03		0.05±0.01	2.14±0.07
L-EVA-1143	210	10±5	20.8±3.3	21.1±1.5	15.5±2.6	26.3±1.2	600±35	1.74±0.05	0.66±0.04		0.04±0.01	2.44±0.17
L-EVA-1083	260	10±5	22.3±3.1	20.6±1.0	14.0±3.8	25.7±1.2	707±17	2.32±0.05	0.75±0.04		0.04±0.01	3.11±0.18
L-EVA-1084	300	10±5	28.9±3.1	21.9±1.0	15.2±4.2	25.2±1.2	554±14	1.87±0.03	0.74±0.04		0.04±0.01	2.65±0.15
L-EVA-1085	375	10±5	22.1±3.1	18.9±0.9	18.3±4.1	24.7±1.1	495±12	1.81±0.03	0.49±0.03		0.04±0.01	2.34±0.13
L-EVA-1144	375	10±5	26.2±4.7	24.8±2.1	21.4±6.2	25.4±1.5	508±38	1.72±0.04	0.49±0.03		0.04±0.01	2.25±0.15
Terrace section												
L-EVA-1145	45	10±5	15.0±2.5	14.9±1.0	9.6±1.8	15.6±0.8	284±18	0.76±0.02	0.33±0.02		0.22±0.03	1.31±0.09
L-EVA-1146	70	5±3	14.9±3.9	15.5±1.2	14.6±2.2	13.7±0.8	197±15	0.79±0.01	0.36±0.02		0.21±0.02	1.36±0.08
L-EVA-1212	100	5±3	9.5±1.5	9.6±0.5	10.0±1.8	8.9±0.5	136±6	0.62±0.01	0.22±0.01		0.21±0.02	1.05±0.04
L-EVA-1213	115	5±3	11.5±1.8	9.4±0.5	15.4±1.6	10.0±0.5	130±7	0.93±0.02	0.27±0.01		0.20±0.02	1.40±0.05
L-EVA-1148	150	5±3	14.2±2.6	15.1±1.0	12.2±1.8	15.3±0.8	242±15	0.81±0.02	0.27±0.01		0.20±0.02	1.27±0.07

^a^Attenuated with respect to the moisture content of each individual sample.

Gamma dose rates based on field gamma-ray spectra, reflecting the in situ radiation geometry of each sample point, and on HRGS show varying degrees of agreement with one another. Given the heterogeneous gamma radiation environments of the majority of samples at Rhafas results from in situ measurements were preferred.

Beta dose rates were calculated using a Risø low-level beta multicounter system GM-25-5 [47, 48]. Dried material from the ends of the OSL-sampling tubes or the outer surface of the block samples was milled to fine powder. About 1.5 g of homogenised sample was placed in each plastic sample holder, covered with cling film (to avoid contamination) and a plastic ring was pressed around the sample holder to hold the assemblage in place. For each OSL sample, four sub-samples and one standard were counted simultaneously for 24 h.

Comparisons between beta dose rates calculated using low level beta counting and HRGS show discrepancies in some samples while for others the results are consistent between methods (S1 Table). In the lower cave section, below Layer 6d, where sediments are comparatively homogeneous, beta dose rates for both techniques are in agreement with one another. In the sedimentologically more complex layers of the cave and in the terrace section, beta dose rate comparisons are more likely to show deviations. As beta particles are only able to travel <1 cm in sediments, we consider the beta dose rates from low-level beta counting on material from the same sampling tube (or block) used for D_e_ determination to be more reliable than HRGS results measured on bulk material of 0.5–1.5 kg of sediment surrounding the OSL sample.

The average moisture content was estimated with respect to both in situ and saturation moisture content. The field moisture values were determined by weighing raw and oven-dried samples. Full-saturation moisture content was estimated as the ratio of weight of absorbed water to dry sample weight. Based on the results, dose rates were calculated assuming average burial-time moisture contents of 5±3% for the cemented and 10±5% for the uncemented layers to account for attenuation [49].

The cosmic dose rate was calculated according to Prescott and Hutton [50] from the altitude and geomagnetic latitude of the site, the burial depth, and the density of the overburden. The results of the dose rate determination are summarised in Table 1.

#### Equivalent dose determination

Luminescence measurements were performed on three Risø OSL/TL readers (DA-15 and DA-20 with single grain attachments), each equipped with calibrated ^90^Sr/^90^Y beta sources [51] and fitted with 7.5 mm Hoya U-340 detection filters [52]. The machines were equipped with infrared diodes (875 nm) and blue light-emitting diodes (470 nm). Green lasers (90% power) emitting at 532 nm were used for light stimulation of single grains [51]. Because only small amounts of material were available after the chemical treatment, standard performance tests on small aliquots (1 mm) were performed using the 125–180 μm in diameter sand fraction. Single grain dating of sand-sized quartz grains (180–212 μm) was used for D_e_ determination. Single grain discs were loaded by sweeping individual quartz grains over aluminium discs each containing 100 holes with a small brush. The single-aliquot regenerative-dose (SAR) protocol based on Murray and Wintle [29, 53] was applied for initial tests and D_e_ determination (Table 2). In addition to the recycling ratio and the recuperation test, which are normally incorporated within a SAR protocol, the OSL IR depletion ratio [54] was applied to detect feldspar contamination.

**Table 2:** Single aliquot regeneration (SAR) protocol for single grains used in this study.

Run	Treatment
1	Dose (except before first run)
2	Preheat (240°C or 260°C for 10s)
3	Optical stimulation with IR diodes for 100s at 20°C (only for last run)
4	Optical stimulation with green laser for 1s at 125°C
5	Test dose
6	Cutheat (200°C or 220°C for 10s)
7	Optical stimulation with green laser for 1s at 125°C
8	Start from top

Preheat temperatures were determined individually for each sample by performing standard preheat plateau tests as well as combined dose recovery preheat plateau tests at seven different preheat steps (160–280°C, three small aliquots were measured per preheat temperature) [53, 55]. S2 Fig shows that a preheat plateau for all temperatures can be observed for sample L-EVA-1146, whereas sample L-EVA-1139 shows more variable results that stabilise at higher temperatures (S2A Fig). Dose recovery ratios are close to unity and are independent of the preheat temperature (S2B Fig). Based on these results, preheat temperatures for D_e_ measurements were set to 260°C or 240°C and cutheat temperatures to 220°C or 200°C, respectively (S1 Table). To gather more information about the luminescence characteristics and the signal reproducibility of each sample, dose recovery tests were performed on a single grain basis.

Early background subtraction was used for D_e_ determination of single grains to minimize the proportions of interfering OSL signal components [56, 57]; initial and subsequent 0.035 s of the decay curve were taken for signal and background integration, respectively. Since not all individual grains yield useful luminescence signals for OSL dating [39, 58], only those passing a set of strict selection criteria–single saturating exponential curve fitting, intersection with the dose response curve, signal >3x background counts, test dose signal error <20%, recycling ratio <20%, recuperation <5%, IR depletion ratio <5%, and D_e_ error <30%—were chosen for analyses. Between 900 and 4100 single grains were measured until at least 50 D_e_ values for each OSL sample passed the rejection criteria [59].

### Sediment and bedrock analyses

Fresh bedrock samples were collected from the three main lithological units (granodiorite, meta-sediments and dolomitic limestone) in the Rhafas area for X-ray fluorescence (XRF) analyses. Material from the dried ends of the OSL sampling tubes and the outer surfaces of the block samples were used for XRF and grain size analyses. All analyses were carried out at the Institute of Geography, University of Leipzig. A subsample from the prominent duricrust at the top of Layer S5 in the terrace section was analysed at the Department of Geography, University of Leicester, for stable-isotope composition of the carbonates and thin section microscopy. Further details on the methods used for sediment and bedrock analyses can be found in S2 File.

## Results

### Cave stratigraphy

**Fig 3:**
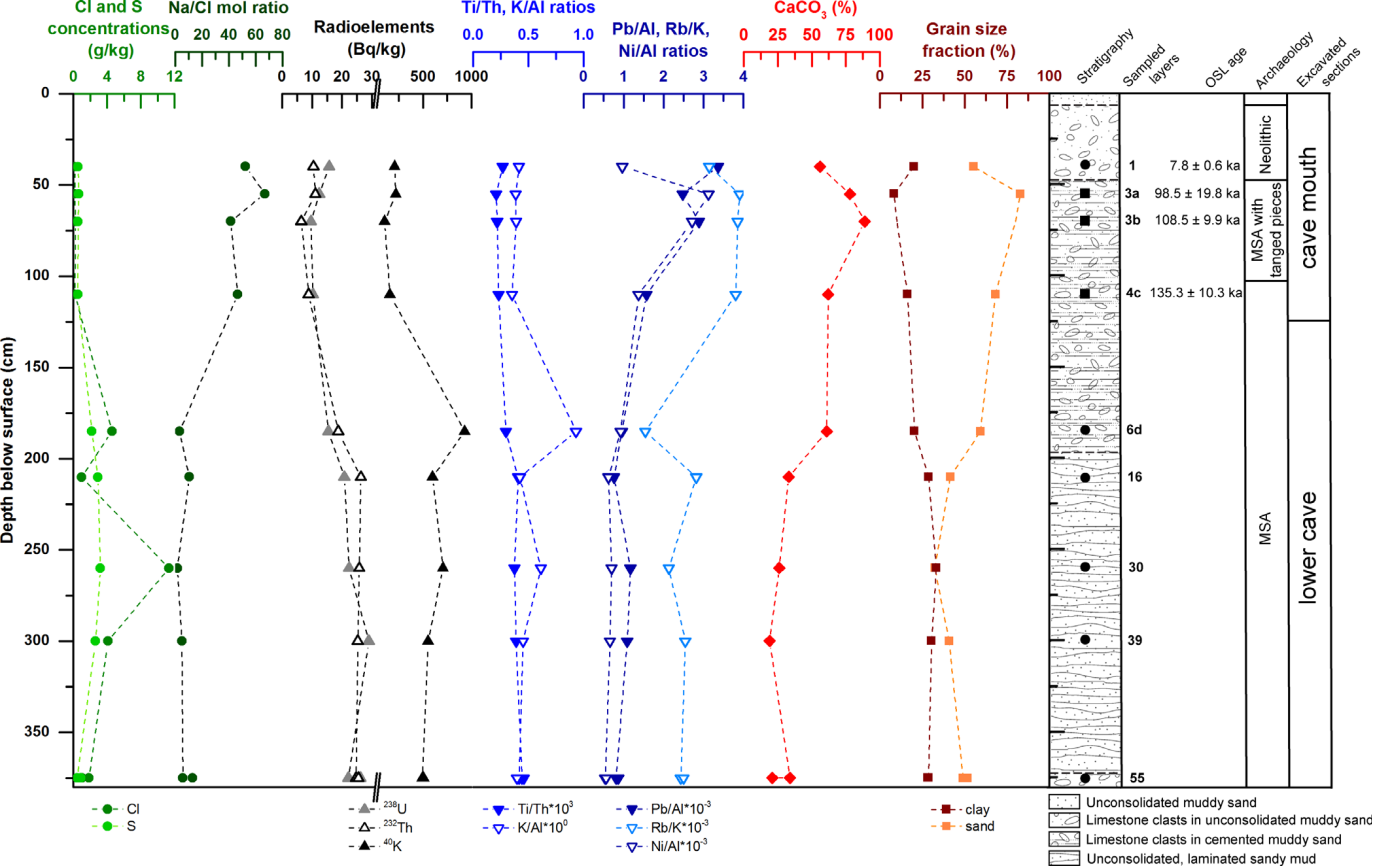
Sedimentological characteristics and stratigraphy of the Rhafas cave deposits. Results of XRF analyses, high resolution gamma spectrometry, calcium carbonate and grain size determination are displayed for each sampled layer from the cave mouth and the lower cave section at Rhafas, together with the determined OSL ages and the corresponding archaeological information.

The sediments of the lower cave section contain assemblages attributed to the MSA and correspond to Wengler’s Unit III [42]. The section is characterised by alternating unconsolidated brown or red sands and silts with interbedded thin calcareous horizons (layers 7–55). Grain size analyses indicate approximately equal proportions for clay, silt and sand in the non-calcareous layers (S5 Table, S5 Fig), and relatively consistent calcium carbonate (CaCO_3_) concentrations (19–33%). Concentrations of Cl and S, which serve as indicators for evaporate enrichment and consequently more arid climatic conditions, vary within the sediments of the lower cave section, while the Na/Cl mol ratio remains homogeneous and close to zero (Fig 3). Layer 6 forms a thick cemented cap on the top of this section and contains limestone clasts in a brown sandy matrix. Both sand and CaCO_3_ content in Layer 6 markedly increase relative to the underlying units to 60% and 61%, respectively (Fig 3). Its cementation further increases in intensity and thickness towards a prominent tufa/flowstone mound on the southwestern wall of the cave, which may represent deposits formed during a period of increased water availability within the cave. The contact between Layer 6 and underlying layers 7–55 is also clearly recognizable from the element concentrations of siliciclastic origin in the XRF data, especially in the K/Al and Rb/K ratios (Fig 3).

The deposits of the cave mouth section are dominated by stratigraphic Units I and II in Wengler’s (1993) framework [42]. The stratigraphic Unit II consists of MSA Layers 2 to 5, while Unit I contains a single layer which shows significant human impact (reworking, ash deposits and archaeological remains) of Neolithic age. The current excavation in the cave mouth did not excavate Layer 2 at all—which appears to have had laterally a rather limited extend—and ends at Layer 4d which forms a prominent floor within the cave (Fig 2B). The layers of the cave mouth section differ substantially from the underlying lower cave section sediments with respect to geochemistry. The siliciclastic ratios of Pb/Al, RB/K and Ni/Al, and carbonate concentrations are higher in the cave mouth sediments than in the underlying units. Likewise, proportions of sand and clay are higher and lower, respectively, in these upper units (S5 and S6 Tables, Fig 3). The sand fraction clearly dominates (up to 83% in Layer 3a) in all layers of the cave mouth section (S5 Fig); CaCO_3_ concentration is also high (up to 90%, also 3a); and there is minimal variability in Cl and S concentrations (<0.6 g/kg). The Na/Cl mol ratio substantially increases (up to 67) in the cave mouth sediments relative to the lower cave section (Fig 3). Layer 3a to 4c show minimal lateral variation and are dominated by angular limestone clasts in a cemented sandy or silty brown matrix. In our excavation area, Layer 1 unconformably overlies Layer 3a and consists of unconsolidated dark grey sediments. The change in sedimentology at the contact between Layers 3a and 1 is reflected by the non-soluble element concentrations with decreasing Pb/Al and increasing Rb/K and Ni/Al ratios (Fig 3).Variations in the Ni/Al and Pb/Al ratios reflect again sedimentologic changes between Layers 3a and 3b (showing a decrease and increase, respectively), as well as 3b and 4c (both ratios decreasing).

The new excavations of the terrace section (Fig 2E) revealed a stratigraphically complex sequence at least ~1.5 m thick. Seven layers were identified (S1 top to S7 base), and these are comprised predominantly of silts and sands alternating with carbonate crusts, the latter most likely the result of post-depositional cementation (S5 and S6 Tables). By the end of the last excavation season in 2010, the base of Layer S7 had not yet been reached.

The sediments of the two lowermost Layers (S6 and S7) were deposited around a field of large limestone boulders, which may be related to an earlier extension of the cave roof, as described in the geological description of the site. These layers contain assemblages attributed to the MSA and are characterised by diagenetic cementation and varying proportions of limestone clasts. Layer S5 is strongly cemented and also contains MSA finds. On top of it, a prominent, finely laminated and several centimetres thick carbonate crust was formed that distinctively marks the contact between S3 and S5. Layer S3 is cemented and comprises reddish grey sediments with abundant limestone clasts. Layer S2 is composed of dark grey ashy sediments with lower carbonate content. Both S2 and S3 yielded lithic assemblages attributed to the LSA, but they are separated by an unconformable contact. S4 displays a cemented conglomeratic facies variation of S3, but it is limited in extent and is most likely associated with cementation of a narrow channel feature.

Though the terrace and the cave sequence are not yet connected physically through excavation, given the lithological and archaeological similarities, it seems likely that terrace Layer S1, consisting of poorly consolidated grey ashy sediments with abundant Neolithic remains and limestone pebbles, correlates with Layer 1 of the cave fill sequence [60]. Layers S2 and S3 contain LSA which is not represented in the cave sequence. Layers S4 through S7 contain MSA with tanged pieces and, therefore, perhaps correlate with Levels 2-3a in the cave sequence. Though the material culture and sedimentological observations provides some general indications in the absence of physically connected stratigraphies, one important additional element for linking the two sections is the robust chronological control described in this paper.

Grain size and geochemical analyses of the terrace section layers show no major variations (S6 Fig), except for the distinctly decreasing Pb/Al ratio between Layer S2 and S3, which is most likely caused by post-depositional, anthropogenic overprint. The values for all sedimentological proxies of the layers from the terrace section are comparable with those obtained for the cave mouth sediments (S5 and S6 Tables). The sand fraction clearly dominates in all layers (54–62%, S5 Fig), while CaCO_3_ content reaches up to 79%. Concentrations of both radioelements and major elements are comparable with the sediments from the cave mouth section.

The bedrock sample from the limestone unit at Rhafas–within which the cave is situated—yields a Ca/Mg ratio of 1.2 and can, therefore, be clearly classified as dolomitic limestone. However, there is no evidence for weathered dolomitic bedrock in the sediments, as Ca and Mg show no significant correlation in the samples (S7A Fig). This suggests that the sediments at Rhafas originate from an allochthonous source.

XRF results of the sediments show negative correlations between Ca and Al and between Ca and Fe (S7B and S7C Fig). The high Ca contents reflect a carbonate-rich sedimentological context at Rhafas, while at the same time the siliciclastic fractions, represented by Al and Fe, are reduced. The Ca correlates positively with the coarse sand (gS) fraction but is relatively insignificant in the silt and fine sand fractions (S7D Fig). This indicates local, secondary carbonate enrichment processes at the site through precipitation of percolating carbonate-rich waters and contradicts an exclusively aeolian origin for the carbonates in the sediments. The highest contents of Ca and gS can be found in the cave mouth and the terrace section, where most layers are presently cemented by carbonates. S7B and S7C Fig illustrate the aforementioned sedimentological similarities between these two sections. Layer 6d, however, shares the same characteristics, which again underlines its exceptional position in the lower cave section.

#### Archaeological context

Four main technological groups were identified in the new excavations. The Neolithic (Layer 1 and S1) is rich in pottery with rare types of cardial ware (A. El Idrissi, pers. comm.) and bone tools (mainly points and some *lissoirs*). The lithic artefacts from Layer S2 and especially S3 in the terrace section show a significant use of microlithis/bladelets, which is characteristic of the LSA [20]. Single platform and opposed platform bladelet cores are common, and the retouched tools are backed bladelets. The MSA (Layers 2 to 55 and S4 to S7) can be separated into two distinct groups defined by the regular occurrence or absence of tanged pieces. Layers 2 to 3b and S4 to S7 contain considerable quantities of tanged pieces and are also characterised by retouched tools such as side scrapers, notches and some end scrapers. A significant Levallois component is present mainly in Layers 2, 3a and S6. In the underlying Layers 3b to 55 tanged pieces do not occur on a regular basis. Large Levallois flakes, laminar flakes, side scrapers and bifacial foliates are common in Layers 4 to 6, whereas only a few such artefacts were recovered from Layers 7 to 51 and Layers 52 to 55 display a variety of side scrapers on Levallois blanks.

Faunal remains are preserved throughout the sequence (S2 Table), although the specimen numbers are small and decrease with depth. Michel [61] provided a list of identified species by layer for the early Wengler excavations, and the assemblages from the recent excavations are being analysed from a zooarchaeological and taphonomic perspective in addition to basic taxonomic identifications. The Neolithic layers include remains of Caprinae (sheep/goat) and Suidae (pigs), which likely derive from domesticated stock, and some Bovinae (cattle/aurochs) remains also likely represent domesticates (continuing work with the faunal aims to investigate this in detail). In addition, the Neolithic assemblages contain a variety of wild taxa, particularly Alcelaphinae (hartebeest/wildebeest), Equidae (horse/zebra), and a few gazelles (*Gazella* sp.). Although overall species diversity is lower in the LSA immediately below, the dominate taxa remain the same, alcelaphines and equids are still most common with a few gazelles and one Barbary sheep (*Ammotragus lervia*) specimen. Sample sizes are too small to provide reliable relative abundances for the older layers, but equids are still most common, and isolated warthog (*Phacochoerus africanus*), Rhinocerotidae (rhinoceros), and Bovinae (aurochs) specimens are present. The persistence of equids through the sequences indicates the consistent exploitation of open, grassy landscapes by the site’s occupants.

#### Duricrust characteristics

**Fig 4:**
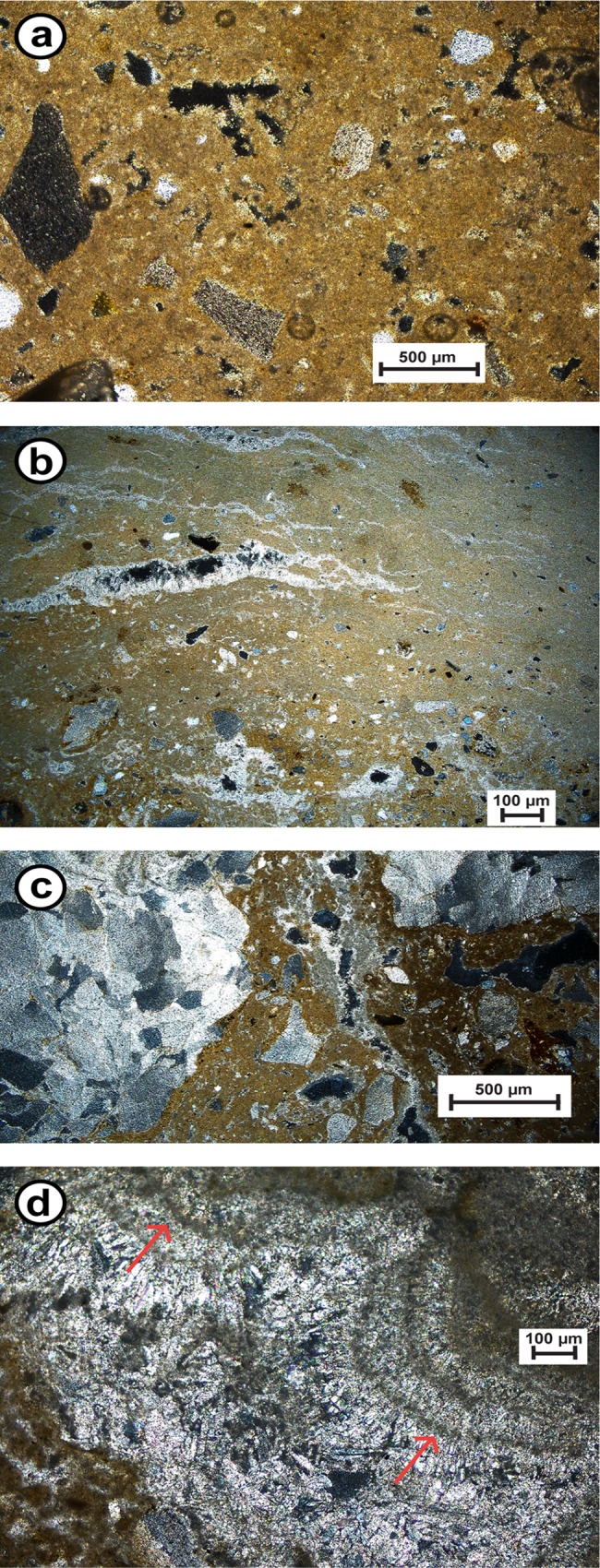
Thin section photographs. (a) Micritic calcrete with displaced semi-rounded lithoclasts and secondary porosity; (b) silcrete-calcrete intergrade duricrusts with silica crystals developed within stringers in the calcrete; (c) calcrete-silcrete intergrade duricrust with clear domination of silica cement over calcrete; (d) silcrete cements: amorphous brown opal followed by fibrous crystals of lussatite, also note the mammillary structured quartz crystals (arrows). All photographs were taken under cross-polarised light.

Thin section microscopy identified the carbonates immediately overlying Layer S5 (S6 Fig) as a diagenetically complex and well indurated duricrust, ranging in composition from calcrete to intergrade duricrusts through to silcrete (Fig 4A–4D). Duricrusts are geochemical sediments that form a zone of accumulation of soluble chemical precipitates within or replace underlying deposits through the movement of mineral-bearing waters [62]. Further detailed descriptions on the various components of the duricrust can be found in S3 File.

Analyses of isotopic compositions were undertaken on subsamples from a well indurated calcrete *sensu stricto* (Fig 4A), as well as from organic layers and a laminar crust (S7 Table), both preserved within the calcrete. Mean values for δ^13^C are -6.14, -2.03 and -9.12 and for δ^18^O -6.39, -8.82 and -5.33 for the calcrete, the organic layer and the laminar crust, respectively (S3 File).

### OSL dating

#### Luminescence signal and equivalent dose characteristics

**Fig 5:**
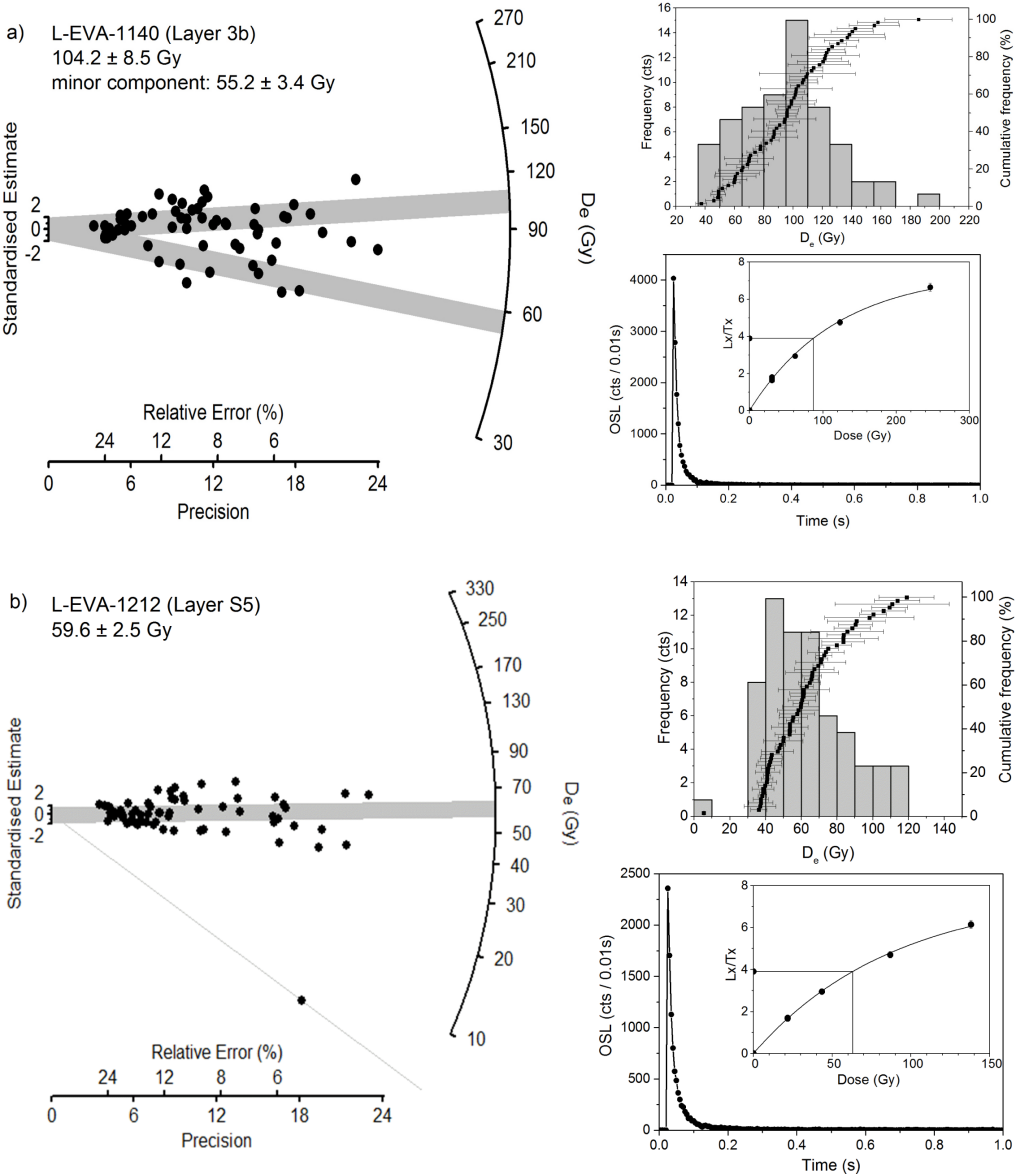
Representative OSL characteristics for two sediment samples from Rhafas. Radial plots and frequency histograms show the dose distributions of single grains, and a natural OSL decay curve with dose response curve (as inset) for samples (a) L-EVA-1140 and (b) L-EVA-1212, respectively. The shaded bands in the radial plots correspond to the standard error deviation from the calculated D_e_.

Individual grains from Rhafas exhibit rapidly decaying luminescence signals typical for fast component dominated quartz (Fig 5, S11 Fig), but only 1.3–7.9% of all measured single sand-sized quartz grains proved suitable for OSL dating using the SAR procedure (S3 Table). Dose recovery tests on individual grains demonstrate the ability of the samples to consistently recover a known laboratory dose within two 2-sigma of unity (S4 Table).

In most instances, the samples from the terrace section yield normal D_e_ distributions (S3A–S3D Fig), characteristic for well bleached and unmixed samples. Overdispersions are below 30% (Table 3), with the exception of sample L-EVA-1212 (Fig 5B), which yields a higher value of 44%. The radial plot of sample L-EVA-1212 shows a one-grain-population (D_e_ ~5 Gy) that clearly separates from all other accepted grains and does not belong to the population of grains representing the depositional age of the sample (Fig 5B). It remains ambiguous whether this single grain was introduced into the layer by post-depositional mixing, which seems unlikely, or was shielded by carbonates during burial time or simply exhibits insufficient luminescence characteristics. This individual grain was considered an outlier and excluded from further analyses even though it was not identified as such by the Grubbs test [63]. As a consequence, the overdispersion for L-EVA-1212 was reduced to 30%. The Central Age Model (CAM) [64], which assumes a single homogeneous age population, was used for age calculation of the terrace section samples.

**Table 3:** Results of OSL dating.

Sample	Unit	CAM^a^	Overdispersion	FMM^b^ D_e_ values (Gy) and proportions (%)				Total dose	Age^c^	Age of minor
		D_e_		Component 1		Component 2		rate		component
		(Gy)	(%)	D_e_	proportion	D_e_	proportion	(Gy/ka)	(ka)	(ka)
Cave mouth section										
L-EVA-1210	1	10.0±0.6	37±4	2.7±0.1	4.8	**10.7±0.5**	95.2	1.29±0.07	7.8±0.6	2.1±0.1
L-EVA-1139	3a	**86.2±2.7**	23±2	71.3±14.1	42.2	**99.4±19.7**	57.6	1.01±0.04	85.4±4.5/98.5±19.8	70.7±14.2
L-EVA-1140	3b	90.5±4.0	33±3	56.7±4.6	23.1	**104.2±8.5**	76.9	0.96±0.04	108.5±9.9	59.0±5.4
L-EVA-1141	4c	116.7±6.6	44±4	55.2±3.4	16.4	**134.0±8.3**	83.6	0.99±0.04	135.3±10.3	55.7±4.2
Terrace section										
L-EVA-1145	S2	**20.2±0.6**	20±2	-	-	-	-	1.31±0.09	15.4±1.2	-
L-EVA-1146	S3	**29.2±1.1**	29±3	-	-	-	-	1.36±0.08	21.4±1.5	-
L-EVA-1212	S5	**59.6±2.5**	30±3	-	-	-	-	1.05±0.05	56.9±3.5	-
L-EVA-1213	S6	**121.4±5.0**	29±3	-	-	-	-	1.40±0.05	86.4±4.9	-
L-EVA-1148	S7	**155.5±6.4**	30±3	-	-	-	-	1.27±0.07	122.5±8.8	-

^a^Central Age Model [64].

^b^Finite Mixture Model.

^c^Calculated using the D_e_ highlighted in bold.

**Fig 6:**
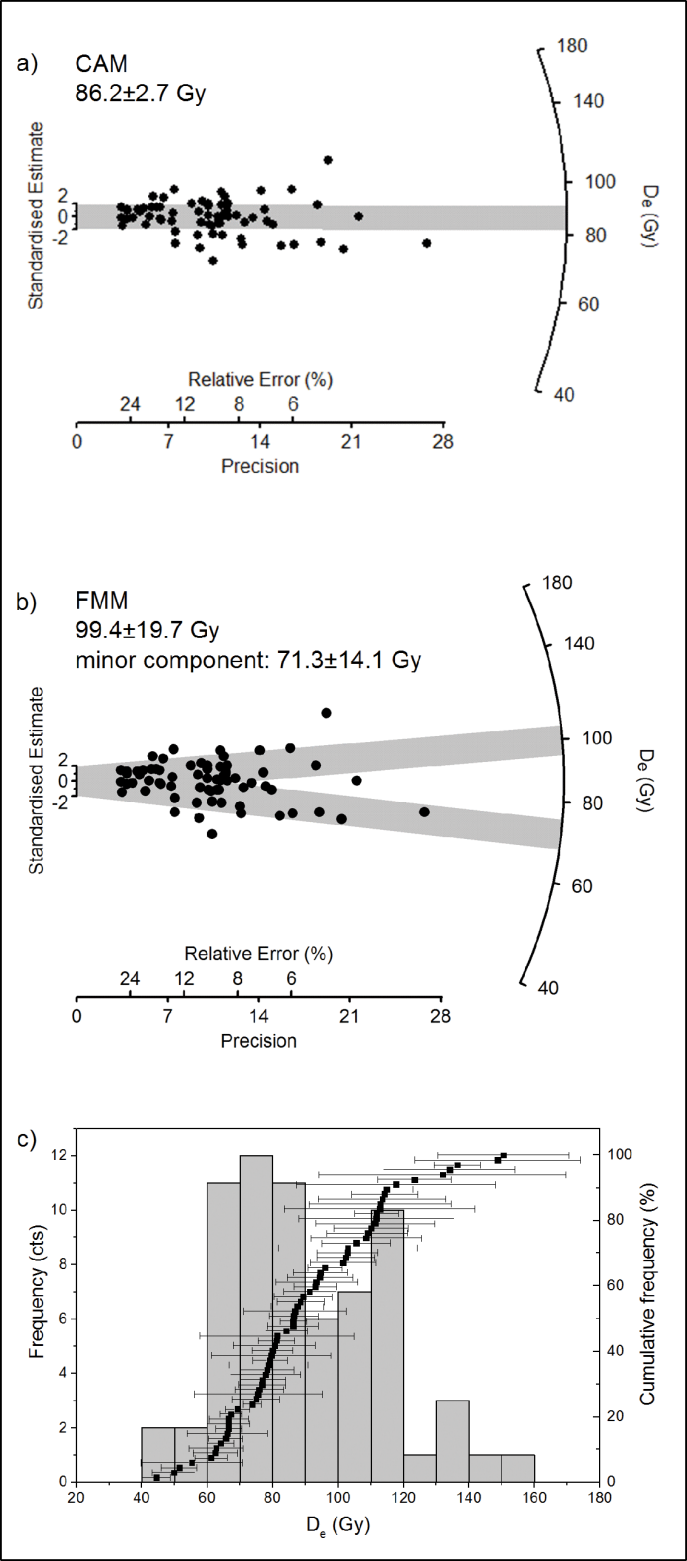
Dose distributions of single grain values obtained for sample L-EVA-1139. The shaded bands in the radial plots correspond to the standard error deviation from the D_e_ calculated using the (a) Central Age Model or (b) Finite Mixture Model. (c) Frequency histogram of single grain D_e_ values.

The samples from the cave mouth section yield generally more widespread single grain distributions with overdispersions ranging from 23 to 44% (Figs 5A and 6; S4 Fig; Table 3). As there is no indication of incomplete bleaching or proportionally high dose rate heterogeneity attributed to the samples, the most likely explanation for the observed spread is the post-depositional introduction of younger grains.

To correctly account for the possibility of multiple depositional and/or mixing phases, in addition to the CAM, the Finite Mixture Model (FMM) [65] was systematically applied to all samples from the cave mouth section (Table 3). We ran the FMM for 2–3 discrete dose components using overdispersion values between 15 and 30% and compared the obtained estimates of the Bayes Information Criterion (BIC) and the values of maximum log likelihood (llik) to correctly assess the minimum number of statistically supported D_e_ components for each sample [65, 66]. The smallest BIC values were obtained when running the FMM with two discrete components and overdispersions of 15% (L-EVA-1139), 20% (L-EVA-1140) and 25% (L-EVA-1210 and L-EVA-1141). A substantial increase of llik (by at least 2) when running the model with three components was not observed [65, 66]. Further details on the determined D_e_ values for all samples, their associated D_e_ errors and the relative proportion of individual grains in each identified component are listed in S8 Table.

The FMM statistically supports two discrete components for each sample of the cave mouth section with the minor components containing, with the exception of sample L-EVA-1139, 5–23% of the total amount of accepted grains, which seems reasonable for post-depositional mixing events. Final ages were calculated based on the FMM results, and for sample L-EVA-1139, which yielded a comparatively low overdispersion value (22%), on both the CAM and FMM results, respectively (Table 3).

#### Dosimetry

The total dose rates of the samples in this study vary substantially, ranging from 0.96±0.04 Gy/ka to 3.11±0.18 Gy/ka (Table 1). Lower dose rates were observed for the sediment layers of the cave mouth section and the terrace section (between ~1 Gy/ka and ~1.4 Gy/ka). Units with large proportions of calcium carbonates at Rhafas (L-EVA-1139, 1140, 1141, 1212) also yielded comparatively low total dose rates.

**Fig 7:**
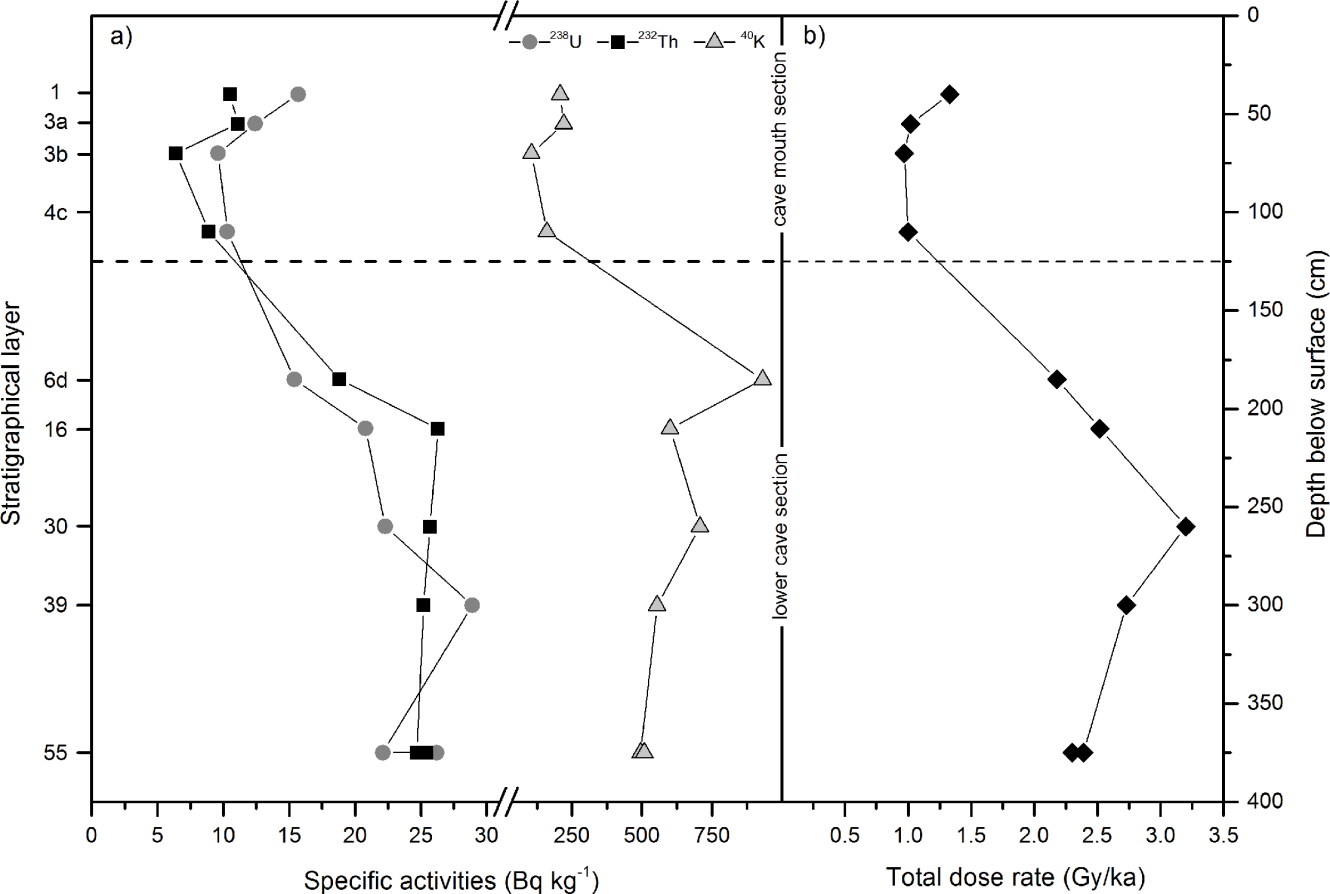
Determined dose rate changes throughout the cave sections at Rhafas. Distribution of (a) radioactive elements ^238^U, ^232^Th, ^40^K and (b) total dose rate for the sampled layers within the cave of Rhafas.

In the cemented and uncemented layers of the lower cave section, dose rates increase significantly for both the beta and the gamma component (Table 1). Concentrations of the radioactive minerals within the sediment layers of the cave mouth section and the lower cave section reflect this growth (Fig 7).

### Chronology

The ages were calculated based on the model-derived D_e_ values outlined in the preceding section and on the dose rate determined for each individual sample. A summary of the obtained values is given in Table 3; the D_e_ values highlighted in bold were used for final age calculations.

The MSA deposits in the terrace section accumulated between 123–57 ka. The two basal samples collected from Layers S6 and S7 (L-EVA-1213 and -1148) give ages of 86±5 ka and 123±9 ka respectively, indicating deposition during the last interglacial (Marine Isotope Stage (MIS) 5). Layer S5 (L-EVA-1212) dates to 57±4 ka and represents an occupation and sediment accumulation phase at the site during MIS 3. The two overlying Layers S3 and S2 (L-EVA-1145 and -1146) give ages of 21±2 ka and 15±1 ka, respectively, and are associated with LSA technocomplexes. The sediment deposition took place during MIS 2, more specifically, during the middle and late stages of the last glacial maximum (LGM).

There is a major erosional break in the cave mouth section separating the Neolithic Layer 1 (L-EVA-1210) deposited ~7.8 ka from the underlying sediments associated with the MSA (Layer 3a to 4c) and deposited >85 ka. The FMM-derived ages suggest deposition of Layer 4c and 3b (L-EVA-1141 and -1140) at 135±10 ka (MIS 6) and 109±10 ka (MIS 5), respectively, and a phase of post-depositional introduction of younger grains between 56±4 and 59±5 ka (MIS 3). For sample L-EVA-1139 (Layer 3a) ages were calculated for both CAM- and FMM-derived D_e_ values. With the CAM, Layer 3a gives an age of 85±5 ka (Fig 6A). The FMM gives ages for the major component (58%) and the minor component (42%) of 99±20 ka and 71±14 ka, respectively (Fig 6B). Both calculated depositional ages for Layer 3a (85±5 ka and 99±20 ka) are consistent with the archaeological finds and date to MIS 5.

## Discussion

### Remarks on dating the lower cave section

Within this study, a substantial dose rate inconsistency was detected between the cave mouth and the lower cave section at Rhafas. Radioactive elements increase substantially from Layer 4c to 6d leading to a rise of the total dose rate by more than a factor of 2 (Table 1; Fig 7). Mercier et al. [26] determined the radioisotopic content for the only OSL sample in their study with a high purity Ge detector, resulting in a total dose rate value of 2.20±0.10 Gy/ka for Layer 6d, which is statistically identical to our data (2.14±0.07 Gy/ka). Dose rates increase with depth in the lower cave section and reach a maximum of 3.11±0.18 Gy/ka in Layer 30 which is approximately 1.35 m below the cave mouth section.

In contrast to the dose rates, D_e_ values in the cave increase continuously with depth without any remarkable deviation. Calculating final ages based on these data would lead to an age inversion, with the lower cave section being younger than the overlying layers of the cave mouth section. Since the D_e_ values for the lower cave section show consistently stable luminescence characteristics and are consistent with the other sections at Rhafas, the aforementioned problems in age calculation most likely originate from the abnormal increase in total dose rates observed in these sediments.

Our sedimentological investigations in the field as well as in the laboratory show no indication of a shift in the main accumulation processes between the two cave sections. The sedimentology of the cave sediments, however, displays major changes in the non-mobile elements (siliciclastic fractions) between the sections which indicate a shift in the sedimentary source over time. This may explain at least part of the increase of radioactive elements (Fig 3). Although the observed differences in the grain size compositions of the layers in the two cave sections most likely reflect primarily the presence or absence of secondary carbonate cementation, the increasing clay content in the lower cave section may support our hypothesis for variability in sediment source. Clay concentrations of 20–30% in a predominantly aeolian cave deposit strongly indicate the presence of a second local sedimentological process which influenced the lower cave section. The clay is either a local weathering product of the dolomitic limestone which forms the cave or reflects past pedogenesis which is no longer preserved in the present-day profile.

Irrespective of clay origin in the lower cave section layers, it does not provide a comprehensive explanation for the substantial increase of potassium observed in these sediments (S10 Fig), and furthermore by the particular peak in ^40^K and correspondingly low clay content within Layer 6d. A possible post-depositional decrease in total dose rates in carbonate cemented layers is also highly unlikely, since the dose rates of unconsolidated layers in the cave mouth and terrace section (1 and S2) are consistent with those of the cemented layers. Moreover, the high (60%) carbonate content in Layer 6d - comparable with the overlying Layer 4c from the cave mouth section–does not appear to influence the downward increase in total dose rates observed between these two layers.

The most likely explanation for the increase in dose rates below Layer 6d - beside the possible influence of a change in sediment source—is a post-depositional input of mobile radioactive elements to the lower cave sediments. Groundwater could easily percolate through the fracture network of the lithological unconformity between the meta-sediments and the dolomitic limestone (located 1–2 m below the cave mouth section), mobilising radioactive elements from the underlying meta-sediments and granodiorite and precipitating them in the lower part of the cave fill sequence. The prominent tufa/flowstone mound on the southwestern wall of the cave clearly indicates spring activity associated with Layer 6d which might have affected the subjacent sediments. While potassium is relatively immobile, it is soluble in water following feldspar weathering [67]. It is readily incorporated into clay mineral lattices, a process which might explain the high ^40^K concentration in Layer 6d relative to the underlying sediment layers.

Unfortunately it is impossible to precisely determine the total amount and timing of precipitation of allochthonous radioactive elements in the individual sediment layers. Moreover, and particularly in the context of a dating study, the timing and flux of fluid activity in the cave is of particular relevance and renders these sediments effectively undateable despite stratigraphically consistent D_e_ values for the OSL samples. Therefore, we regard the age estimate for Layer 6d published by Mercier et al. [26] as an underestimate, and argue that, without further investigations, the lower cave section at Rhafas remains undateable.

### Chronology of deposition

**Fig 8:**
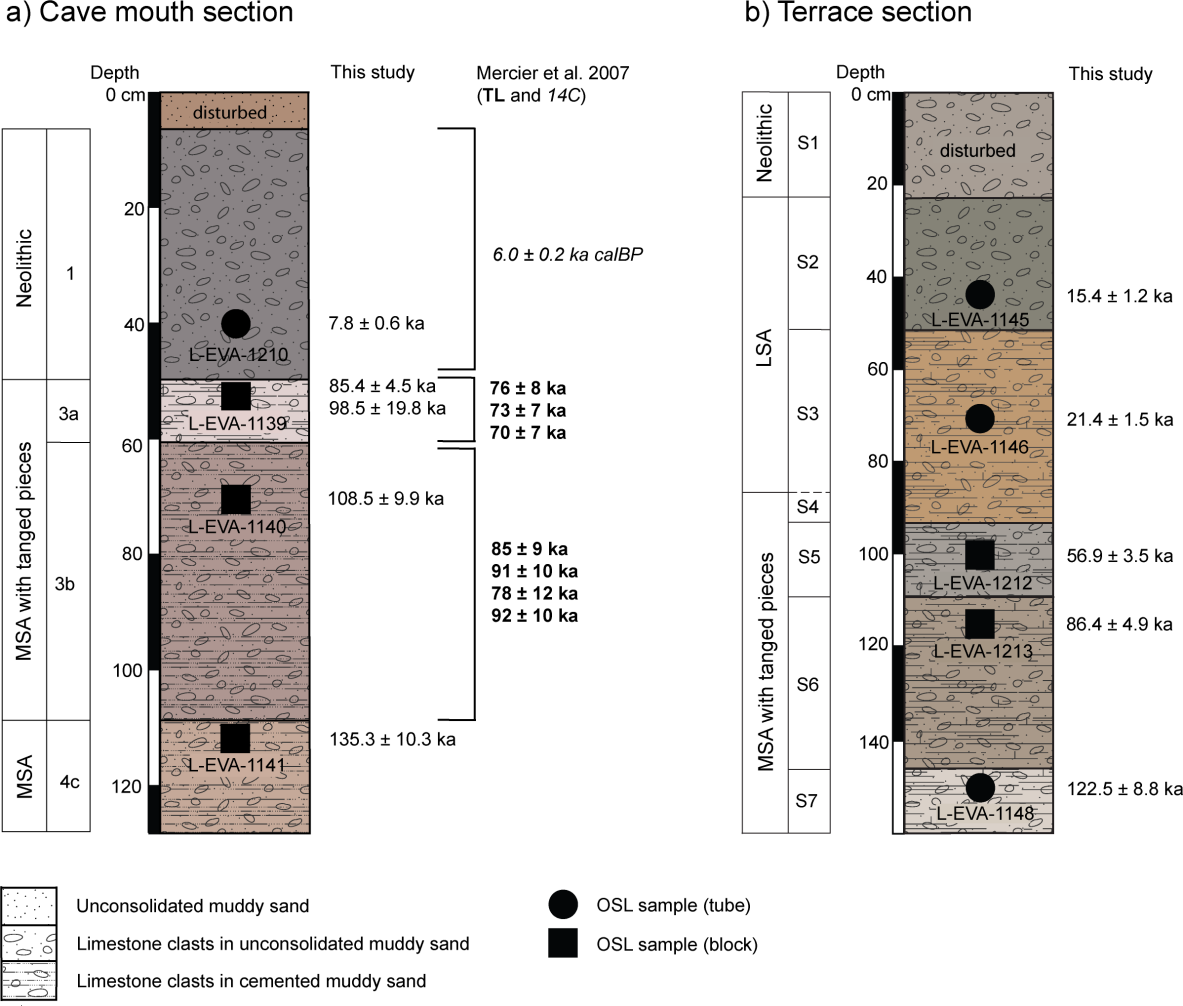
Chronostratigraphy of Rhafas. Determined single grain OSL ages and formerly published absolute age estimates by Mercier et al. 2007 [26] plotted against the stratigraphy of (a) the cave mouth and (b) the terrace section at Rhafas.

Fig 8 summarises the chronostratigraphy of the cave mouth and the terrace section at Rhafas based on single grain OSL age estimates obtained in this study, in comparison with previously published data by Mercier et al. [26]. Deposition of the sediments in the cave mouth section took place between 135 ka and 7.8 ka with a major unconformity between the MSA and the overlying Neolithic (Fig 8A). Although our OSL ages for the MSA Layers 3a and 3b are both slightly older than the corresponding TL age estimates by Mercier et al. [26], both datasets are consistent with each other within the given error ranges as well as with the expected age of the archaeological finds based on chronologies from other sites. OSL and ^14^C age determinations for the Neolithic Layer 1 yielded results of 7.8±0.6 ka and 6.0±0.2 ka cal BP, respectively, which fall into the expected age range.

There is evidence within the three cemented Layers 3a, 3b and 4c for a post-depositional phase where younger material was incorporated into those units around 56 ka. This process involved infiltration of younger grains into the older, still unconsolidated sediment layers, in diminishing concentrations down the stratigraphic profile as indicated by the proportion of younger grains within each sample. FMM analysis of the single grain D_e_ distributions clearly shows the presence of two age populations–an older, dominant population and a younger, minor population—in both Layers 3b and 4c (L-EVA-1140 and -1141). Indications of younger sediment infiltration into Layer 3a (L-EVA-1139), by contrast, are not as unequivocal, and consequently ages for Layer 3a were calculated using both CAM- and FMM-derived D_e_ values in order to interrogate the data before final interpretation of the age (Table 3). The CAM assumes a Gaussian single grain D_e_ distribution [64], and this holds for L-EVA-1139 and is further supported by its 23% overdispersion (Fig 6A). Layer 3a gives a CAM age of 85±5 ka. The FMM-derived D_e_ values, in comparison, result in major (58%) and minor component ages (42%) of 99±20 ka and 71±14 ka respectively (Fig 6B). The relatively similar proportions, and large uncertainties, of the two components of sample L-EVA-1139 can be used to argue that the FMM cannot clearly distinguish two discrete age populations and, therefore, that the FMM is unsuitable. On the other hand, when the dating results from the two underlying Layers 3b and 4c are taken into consideration, there may have been one single post-depositional mixing event affecting all three layers at the same time (~56ka), since the proportions of the minor, younger age component—representing the mixing event—decrease from top (3a) to bottom (4c) and lie within the same age range (Table 3; S9 Fig). Given this interpretation, the FMM is in fact the most parsimonious age model for Layer 3a; the relatively similar proportions and large uncertainties of the two FMM components for this sample are most likely caused by the depositional events occurring within short temporal succession. There are doubtlessly strong arguments supporting the application of either the CAM or the FMM; however, as stratigraphic indications should be fundamental for any dating study, we consider the FMM age to be more conclusive. Regardless of this discussion, both models provide ages that are consistent with the archaeological finds as well as the cave stratigraphy and date to MIS 5.

The earliest deposition of sediments in the terrace section is dated to ~123 ka (Layer S7, Fig 8B). These sediments form a matrix between large dolomitic limestone boulders. The boulders are interpreted to originate from a former extension of the cave roof prior to 123 ka. The stratigraphy of the terrace section sediments is mostly undisturbed (except for Neolithic Layer S1) with no indications for post-depositional mixing or major unconformities. The most remarkable feature of this section is the prominent indurated duricrust that separates Layer S5 (~57 ka, MIS 3) from the overlying Layer S3 (~21 ka, MIS 2) and consists of numerous sublayers of calcretes, intergrade duricrusts and silcretes. The timing of duricrust formation must, therefore, fall in the range 57–21 ka. Since the age of post-depositional sediment incorporation into Layers 3b and 4c at the cave mouth section also dates to ~56 ka, during which time the layers were still sufficiently unconsolidated to allow infiltration of younger grains, pedogenesis (including carbonate induration) of those layers must postdate this process.

### Environmental implications

Regionally, central North Africa has been dominated by C_4_ vegetation and arid conditions over the last 190 ka, with some expansion of C_3_ vegetation assemblages during wetter climatic phases [68]. Rhafas experiences a semi-arid Mediterranean climate (300 mm rainfall p.a.) and lies ~50 km from the present-day coastline (indicating a slight marine influence on both δ^13^C and δ^18^O values), resulting in a mixture of C_3_ and C_4_ plants. Westerlies currently bring winter rains to North Africa and are mainly controlled by the North Atlantic Oscillation [69, 70]. Summers are dry and hot due to the influence of the subtropical high pressure belt [69, 71]. Today, summer rains associated with the African or Indian monsoon do not penetrate far enough north to provide rain to the Sahara or the Maghreb. Climatic conditions in North Africa were, however, substantially different during “green Sahara” events when intensification and northward migration of the monsoonal systems led to enhanced humidity and expansion of subtropical savannah landscapes in the region [72]. While these “green Sahara” events were restricted to relatively short (<5–10 ka) time intervals, past glacial phases were characterised by comparatively cool and arid climatic conditions and interglacials by warmer average temperatures and enhanced monsoon rains. The long term climatic trend over the past several hundred thousand years in North Africa appears to be one of increased aridity [69, 72–74].

Our dating results show that sediment deposition and human occupation at Rhafas initiated at least in MIS 6, when climatic conditions were relatively dry in North Africa, interrupted only by a “green Sahara" event around 170 ka [72]. The sediments of the lower cave section predate 135 ka and show indications for evaporite enrichment (increasing Cl and S concentrations and a decreasing Na/Cl mol ratio, Fig 3). This most likely reflects deposition of the lower cave sediments during relatively arid climatic conditions for the interval between 165 and 140 ka. This time period is also associated with an increased dust flux in the Sahara [72].

While the geochemistry of the sediments from the cave mouth and terrace section share the same characteristics, major differences can be observed in comparison to the lower cave section sediments (Fig 3, S6 Fig), especially in the siliciclastic fractions. Ti/Th, K/Al, Pb/Al, Rb/K and Ni/Al ratios reflect concentrations of primary non-soluble elements, which are unlikely to get introduced into sediment layers post-depositionally. The substantial changes in the siliciclastic ratios between the different sections at Rhafas can, therefore, be attributed to a change in sediment source towards the end of MIS 6/beginning of MIS 5. As north-western Morocco was primarily influenced by the westerlies, this change in sediment source is most likely linked to a shift in wind directions on a regional or local scale.

During MIS 5, climatic conditions were characterised by increasing humidity. “Green Sahara” events took place during substages 5a, 5c, with a particularly strong event around 125 ka (MIS 5e). Studies have shown that the north-eastern Sahara received at least 500 mm of annual rainfall during MIS 5e which enabled the widespread occurrence of wooded savannah landscapes inhabited by subtropical fauna [75, 76]. These conditions would also have facilitated human expansion across northern Africa and the Levant. With the end of the green Sahara event in MIS 5a and the beginning of MIS 4, climatic conditions became drier, leading to a proposed depopulation of the Sahara after 80 ka [70, 77]. Humans rapidly migrated into desert ecological refugia, coastal areas and sub-Saharan Africa [72, 75, 76, 78]. While MIS 5 sediments are present at Rhafas within the cave as well as on the flat terrace area in front of the cave entrance, including archaeological finds that prove intensive human occupation of the site between ~123 ka to ~86 ka, deposits from MIS 4 are not preserved throughout all section profiles. It is unlikely that sedimentation at Rhafas stopped during MIS 4, since glacial periods are commonly associated with increased aeolian reactivation and deposition [72]; rather, we propose that the absence of MIS 4 deposits is more likely linked to natural or anthropogenic erosion that predates the sedimentation of Layer S5 in the terrace section (~57 ka).

Humidity increased again during MIS 3 in North Africa but did not reach the intensity of MIS 5. Humid conditions intensified between ~45 ka and 30 ka. At this time, the western Sahara was covered by steppe-prairie vegetation [78–80]. Deposits from MIS 3 are present at Rhafas in the terrace section (Layer S5) as well as in the cave mouth section. Although almost all material was removed from the latter at some point after deposition, its former presence is still traceable as minor components that were mixed into the underlying sediment layers (3a-4c).

A prominent duricrust was formed on top of Layer S5 in the terrace section (S6 Fig). To obtain further information on the prevailing environmental conditions during their precipitation, stable isotope analyses of carbon and oxygen were undertaken on three distinct types of calcretes–micrite matrix, calcrete rich in organics and laminar calcrete (Fig 6A). The oxygen isotope ratios in the calcretes indicate a lack of significant evaporation, and the δ^13^C mean value of -6.14 suggests a mix of C_3_ and C_4_ plants comparable with present day conditions. As there is no strong degree of covariance between δ^13^C and δ^18^O, it is unlikely that the two sets of isotope ratios were controlled by the same environmental factor, which would have been an indicator for arid environments [81]. Thus it is more likely that the calcretes formed under semi-arid or seasonally arid conditions. The combined results of the duricrust fabrics, isotope ratios from the duricrust (S7 Table) and the abundance of organic matter are consistent with pedogenic calcretes. The δ^13^C values from the dark organic layers within the calcrete—comprising carbonate, organic matter and oxides—suggest a greater abundance of C_4_ plants (-2.03), indicating more arid conditions during its formation. The equivalent δ^18^O values are depleted which suggests low evaporation rates (a key sign of aridity), and therefore appears to contradict the δ^13^C results from the same material. The δ^13^C value (-9.12) of the laminar crust indicates an increased proportion of C_3_ plants and perhaps more of a Mediterranean environmental influence.

Duricrust formation must have occurred between the deposition of Layer S5 (~57 ka) and S3 (~21 ka) during MIS 3 and MIS 2, respectively. The western Mediterranean climate during MIS 2 was characterised by semi-arid to arid conditions [82]. The faunal remains from Layer S3 at Rhafas suggest the presence of open, grassy landscapes at that time.

While humid conditions in northern Africa during MIS 3 might have been favourable for the formation of silcretes, calcretes on the contrary tend to form in semi-arid to arid, but not hyper-arid, conditions. The characterisation of the various components of the duricrusts, supported by the isotope results of the calcretes, suggest that the duricrust at Rhafas is most likely a product of formation under the range of climates that north-western Africa experienced over the last 57 ka rather than of one single climatic event of short duration.

During the early-middle Holocene (10–6 ka) another green Sahara period with increased humidity and expansion of savannah landscapes took place in northern Africa [72, 74]. Sediment deposition at Rhafas associated with the occupation of the site by Neolithic groups date to ~7.8 ka.

### Archaeological chronologies and human occupation phases in the Maghreb

The new series of excavations undertaken in the terrace area in front of the cave of Rhafas yielded archaeological find horizons associated with the LSA and thereby help fill the occupational gap between the MSA and the Neolithic formerly reported for the site by Wengler [42]. Rhafas now represents one of the few sites in the Maghreb which provides a well dated archaeological sequence spanning multiple Palaeolithic technocomplexes (Fig 8).

The chronology presented here does suggest occupation of both the terrace and cave during the MSA with tanged pieces, with depositional ages for Layer 3a and S6, and 3b and S7 overlapping in time. The Neolithic Layers 1 and S1 also allow clear correlations between the two sections at that time. Layers from the LSA, while present in the terrace area, are either not present or have been removed from the cave mouth section.

**Fig 9:**
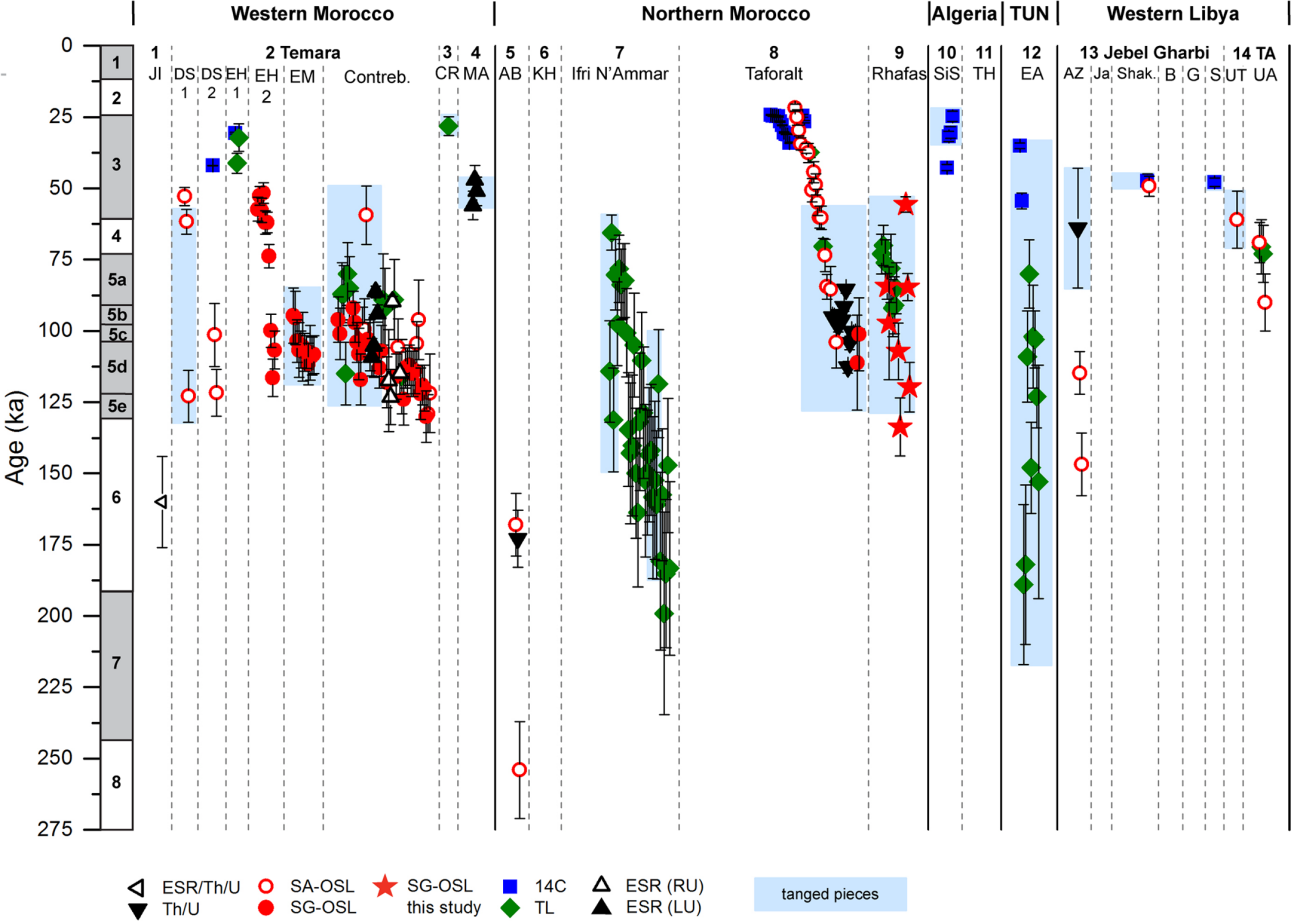
Synthesis of age estimates for MSA layers from archaeological sites in the Maghreb. Numbers in the headline correspond to numbers of the sites given in Fig 1.

**Fig 10:**
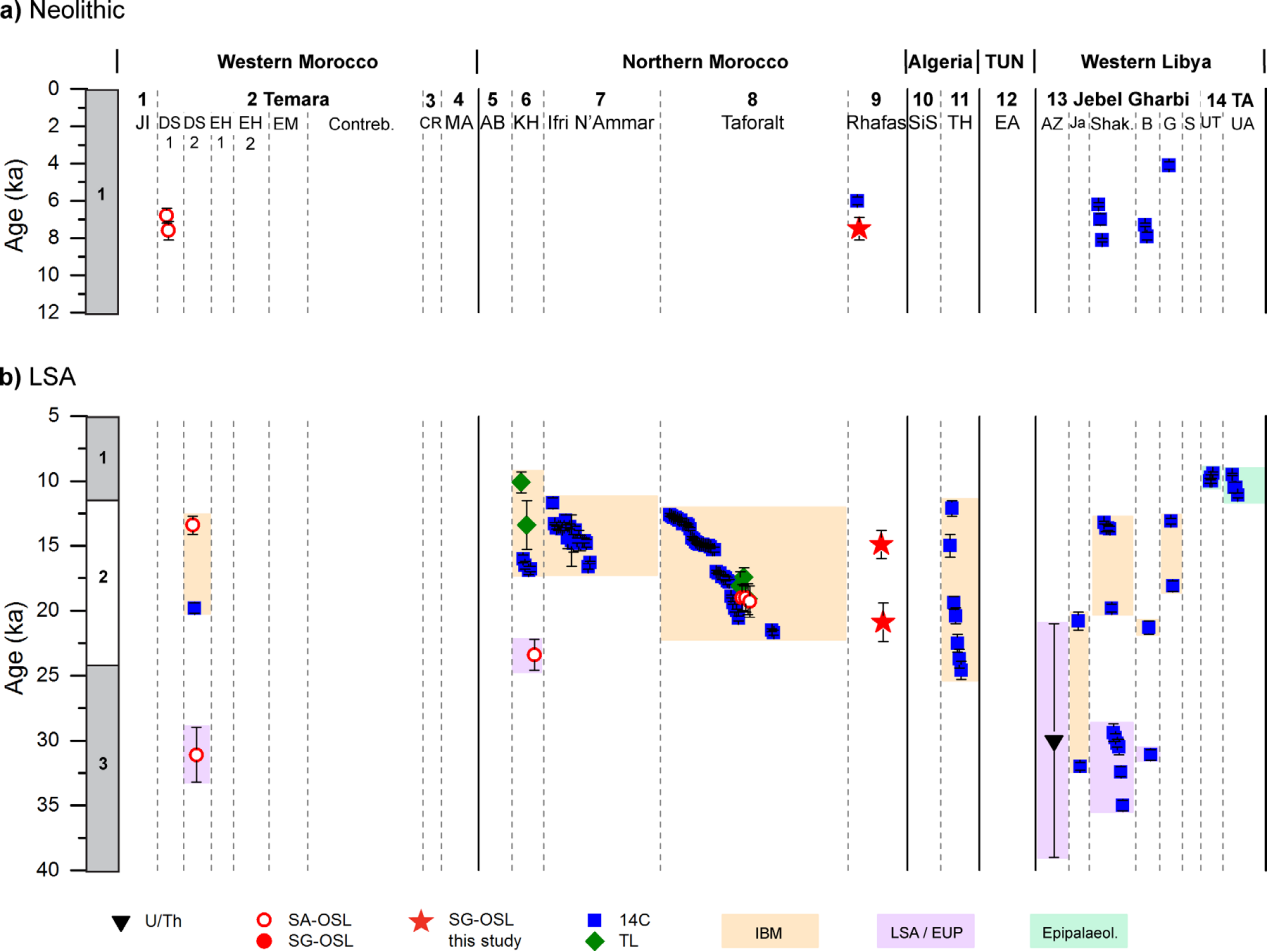
Synthesis of age estimates for Neolithic or LSA layers from archaeological sites in the Maghreb. (a) Neolithic and (b) LSA. Numbers in the headline correspond to numbers of the sites given in Fig 1. Highlighted are dates associated to the Epipalaeolithic, the Iberomaurusian and the LSA/EUP in green, orange and purple, respectively.

Our revised chronostratigraphy from Rhafas contributes towards a rigorous investigation of evidence for human activity across the Maghreb during the Middle and Late Pleistocene. Here we compare our results with published chronological data from 25 MSA and LSA sites across western Morocco, northern Morocco, Algeria, Tunisia and western Libya (Figs 9 and 10; for site locations see Fig 1) [2, 3, 6, 10, 13–17, 26, 28, 31, 33, 34, 83–99]. In this analysis we consider only archaeological layers which can be clearly associated to a defined stone tool technology and have been reliably dated by absolute dating (^14^C, TL, single aliquot OSL (SA-OSL), single grain OSL (SG-OSL), electron spin resonance (ESR) or U-series (Th/U)).

Our data corroborate previously published data and are used to catalyse a synthesis for stone tool assemblage chronologies across the Maghreb.

The first phase of occupation at Rhafas associated with the MSA started at least in late MIS 6 (~134 ka) or even earlier (since MSA is also found in the as yet undateable lower cave section). Its duration was at least 60,000 years and persisted into MIS 5 (Fig 9). Apart from Rhafas, evidence for occupation in the Maghreb in MIS 6 or earlier is limited to the sites of Jebel Irhoud [83], Abrigo de Benzù [31, 33], Ifri N’Ammar [10], El Akarit [92] and Ain Zargha in the Jebel Gharbi [95]. The oldest age is 254±17 ka, recorded at Benzú [31, 33]. The onset of the MIS 5 interglacial resulted in increasingly humid climatic conditions associated with an expansion of grassland habitats in northern Africa [68, 100–102]. These conditions most likely facilitated an increase in human populations living in the region [1]. Occupation during the MIS 5—associated with the MSA—has been published for all parts of the Maghreb, with the exception of Algeria for which no absolute dates are available before MIS 3 (Fig 9). Particularly striking is this phase of settlement at the Atlantic coast of Morocco (Témara region) [3, 13, 14, 16, 17], the sites of Ifri N’Ammar [10], Taforalt [2, 6, 15, 103] and Rhafas in northern Morocco (this study; [26]), and at El Akarit in Tunisia [92].

There appears to be a hiatus in occupation at Rhafas during MIS 4. This may reflect less favourable, drier climatic conditions. However, during MIS 3 occupation associated with the MSA is evident across all areas of the Maghreb.

Some of the key questions relating to the MSA of North Africa refer to the Aterian, its characteristics, technological definition and timing (e.g. [8, 9]). The Aterian has been used to argue for cultural modernity associated with the dispersal of anatomically modern humans out of Africa [4, 6, 7]. There remains some debate over how to define and recognize the Aterian, particularly for MSA assemblages post-dating the earliest Aterian but lacking tanged pieces. There are also assemblages in need of renewed study in light of more recent studies on the non-tanged elements in Aterian assemblages. Consequently, we focus here on the least ambiguous indicator for the Aterian–namely tanged pieces–as an indicator of its temporal onset (Fig 9), while acknowledging that an Aterian without tanged pieces could be yet earlier Dates from archaeological layers containing regular occurrence of tanged pieces have been highlighted in blue (Fig 9). It is important to note that the ^14^C dates of Betrouni [89] from Sidi Sa should be treated with caution. Likewise, the older TL ages from El Akarit may not be reliable due to problems with dose rate determination within the sediments [92]. The earliest reliably occurrence of tanged pieces is found at Ifri N’Ammar, and dated by TL to a weighted average of 145±9 ka [10]. Apart from Ifri N’Ammar, tanged pieces do not regularly occur within Moroccan archaeological assemblages until the onset of MIS 5e ~130 ka [2, 3, 6, 13–17, 26]. Tanged pieces occur even later in Algeria and western Libya: 43±1 ka at Sidi Sa [89], 64±21 ka at Ain Zargha [93], ~47 ka at Jebel Gharbi [93, 94] and 61±10 ka at Uan Tabu [98]. This may be, however, an artefact of the limited chronological dataset for sites in Algeria, Tunisia and western Libya. Tanged pieces persist in archaeological assemblages until at least ~47 ka in western Morocco (Mugharet el’Aliya [86]), northern Morocco (Rhafas, this study) and western Libya (Shakshuk [94]), and maybe until ~35 ka at El Akarit (Tunisia [92]) and ~25 ka at Sidi Sa (Algeria [89]). It is important to note that despite the widespread distribution of tanged pieces within the MSA across the Maghreb after 130 ka, other sites in the region have archaeological layers which do not contain tanged pieces (e.g. El Harhoura 1 [84, 85] and Taforalt [2]).

The youngest dates for the MSA overlap with the onset of the LSA between 30 and 35 ka (Fig 10B). The early LSA is particularly widespread in the archaeological assemblages of western Libya [93, 94]. By contrast, with the exception of Dar es-Soltan II [14], LSA records are not present in other parts of the Maghreb before ~24 ka [34, 91]. At Rhafas, the LSA Layers S2 and S3 date to 15±1 ka and 21±2 ka, respectively, and are consistent with the overall LSA dataset for the Maghreb. Here we follow Linstädter et al. [104] in dividing the LSA of the Maghreb into an early LSA (or EUP), the Iberomaurusian (IBM) and the Epipalaeolithic, as indicated in purple and orange and green in Fig 10B. This recent work at Rhafas has highlighted the exceptional nature and preservation of the LSA marked by one of the earliest appearances of the microlithic bladelet industries in North Africa. The earliest phase of the LSA (Layer S3) is characterised by microliths, bladelets and backed tool industries that occur in Algeria and Morocco around 20 ka [2, 91, 105] and are defined as Iberomaurusian. Elsewhere in Cyrenaica similar technology at Hua Fteah was dated to ~17 ka cal BP [106]. The late phase of the LSA at Rhafas (Layer S2) is typified by a high proportion of backed bladelets and the use of the microburin technique that also occur elsewhere in Algeria [107, 108] and Morocco [109].

The LSA ends with the onset of the Neolithic during the Holocene. In Fig 10A Neolithic age estimates from the MSA and LSA sites in the Maghreb are shown. The dates for Layer 1 from Rhafas fit within the commonly determined age range for the Neolithic and are consistent with the dates from Dar es-Soltan I in western Morocco [13, 14] and the Jebel Gharbi [93, 94]. However, there is still an age gap between the late LSA and the Neolithic at Rhafas. To further investigate the occupation history of the site and the palaeoenvironmental context of the late LSA and Neolithic, additional dating work focusing on the younger occupation phases should be conducted in the future.

## Conclusion

This study presents a revised chronostratigraphy of the archaeological sequence at Rhafas spanning the MSA through to the Neolithic. We used a multidisciplinary approach, applying single grain OSL dating which elucidated post-depositional processes in several stratigraphic units (Layers 3a, 3b and 4c), combined with stratigraphic observations and sedimentological and geochemical analyses of sediments and duricrusts to investigate palaeoclimatic conditions. Our methods were placed within a classical archaeological framework to examine human-environmental interactions at the site during its long occupational history. Our results suggest changes in environmental conditions from arid to humid some time before 135 ka and a phase shortly after 57 ka that favoured carbonate cementation of parts of the archaeological sequences at the site. Detailed investigations of a duricrust layer from the terrace sequence demonstrated their pedogenic origin as well as variability in the climate of north-western Africa since MIS 3. The earliest readily dateable evidence for occupation of the site extends to MIS 6 (~135 ka); the lower deposits, containing comparable stone tool assemblages, remain undateable. Tanged pieces first occur at Rhafas at ~123 ka and continue until ~57 ka, which is consistent with the chronological dataset of the Maghreb for the Aterian. Archaeological layers associated with the LSA are dated to ~21 ka and ~15 ka; although it is unclear whether the site was periodically or continuously occupied during this phase, the nearby site of Taforalt suggests that the latter is possible [2].

## Supporting Information

S1 Fig Parent-daughter equilibrium plots for Rhafas. (a) ^238^U/^226^Ra (b) ^226^Ra/^210^Pb. In each figure the solid line represents secular equilibrium. Samples from the lower section, the cave mouth section and the slope section are shown as black dots, grey squares and open triangles respectively. Dashed lines represent 20% and dotted lines 50% of equilibrium.
(TIF)

S2 Fig Results of OSL standard performance tests. (a) Preheat plateau test (b) Dose-recovery preheat plateau test for samples L-EVA-1139 (grey diamonds) and L-EVA-1146 (black circles) are shown. The solid line indicates the target value; dashed lines represent 10% deviation from unity.
(TIF)

S3 Fig Radial plots showing the dose distributions of single grain values of samples from the terrace section. (a) L-EVA-1145, (b) L-EVA-1146, (c) L-EVA-1213 and (d) L-EVA-1148. The shaded bands in the radial plots correspond to the standard error deviation from the calculated De.
(TIF)

S4 Fig Radial plots showing the dose distributions of single grain values of samples from the cave mouth section. (a) L-EVA-1210 and (b) L-EVA-1141. The shaded bands in the radial plots correspond to the standard error deviation from the calculated D_e_ for each identified component.
(TIF)

S5 Fig Grain-size distributions of the OSL samples per section. Each stacked bar shows the percentage distribution of the grain-size classes for one sample (in total 100%).
(TIF)

S6 Fig Sedimentological characteristics of the terrace section. (TIF)

S7 Fig XRF results showing correlations between Ca and Mg, Al and Ca, Fe and Ca, and Ca and gS. Layer 1 (blue square) and 6d (red dot) plot relatively far away from their groups, which indicates that they do not share the same sedimentological characteristics with the other layers of the cave mouth and the lower cave section, respectively.
(TIF)

S8 Fig Thin section photographs. (a) micritic cement with corroded lithoclasts: around one of the clasts there is evidence of the dissolution of some of the calcite cement and replacement by silica cement (arrow); (b) stringer within the silcrete-calcretes showing the chalcedony crystals in greater detail; (c) preservation of the form of a replaced calcite shell by silicification; (d) mammillary structured quartz crystals showing micro-laminated opal as well as fibrous lussatite and chalcedony. Photographs were taken under cross-polarised light.
(TIF)

S9 Fig Single grain OSL ages for the cave mouth section at Rhafas determined by applying the FMM. Major and minor age components are shown in green and red, respectively
(TIF)

S10 Fig Correlation between ^40^K and clay content at Rhafas. (TIF)

S11 Fig LM-OSL curve for L-EVA-1083, showing dominant OSL fast component. (TIF)

S1 File MSA/MP nomenclature in the Maghreb. (PDF)

S2 File Sediment and bedrock analyses. (PDF)

S3 File Duricrust characteristics. (PDF)

S1 Table Measured moisture contents, beta dose rates and chosen preheat/cutheat temperatures. (PDF)

S2 Table Summary of faunal remains. (PDF)

S3 Table Single grain characteristics. (PDF)

S4 Table Single grain dose recovery properties. (PDF)

S5 Table Grain size results. (PDF)

S6 Table XRF results and CaCO_3_ content. (PDF)

S7 Table Summary of istope data. (PDF)

S8 Table FMM details. (PDF)
