# Supplementary figures and images for: A New Chronology for Rhafas, Northeast Morocco, Spanning the North African Middle Stone Age through to the Neolithic
Source: PLoS One. 2016 Sep 21;11(9):e0162280. doi: 10.1371/journal.pone.0162280 (PMC5031315; doi:10.1371/journal.pone.0162280)

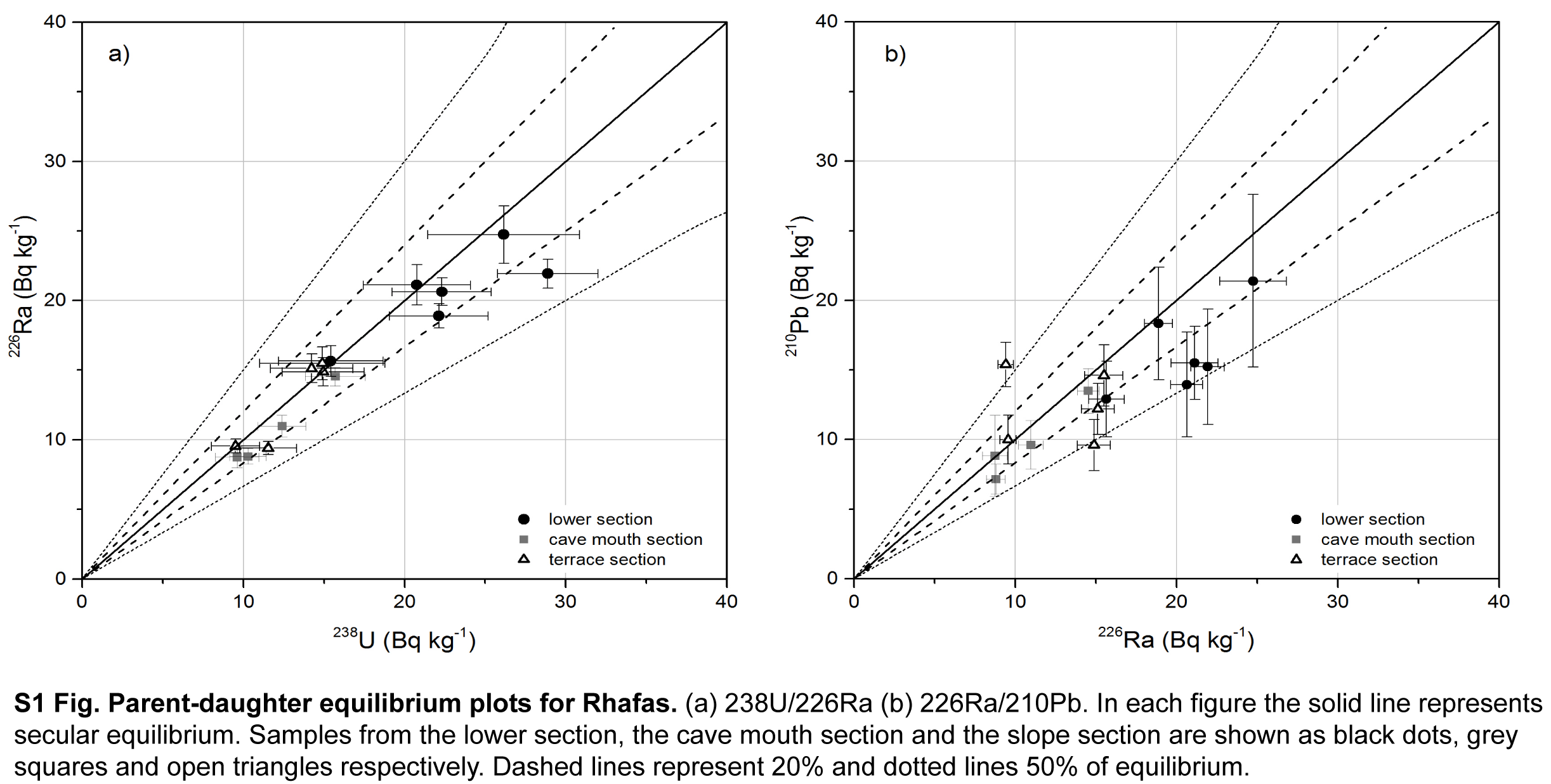

Supplement: S1 Fig — (a) 238U/226Ra (b) 226Ra/210Pb. In each figure the solid line represents secular equilibrium. Samples from the lower section, the cave mouth section and the slope section are shown as black dots, grey squares and open triangles respectively. Dashed lines represent 20% and dotted lines 50% of equilibrium. (TIF) [file pone.0162280.s001.tif]

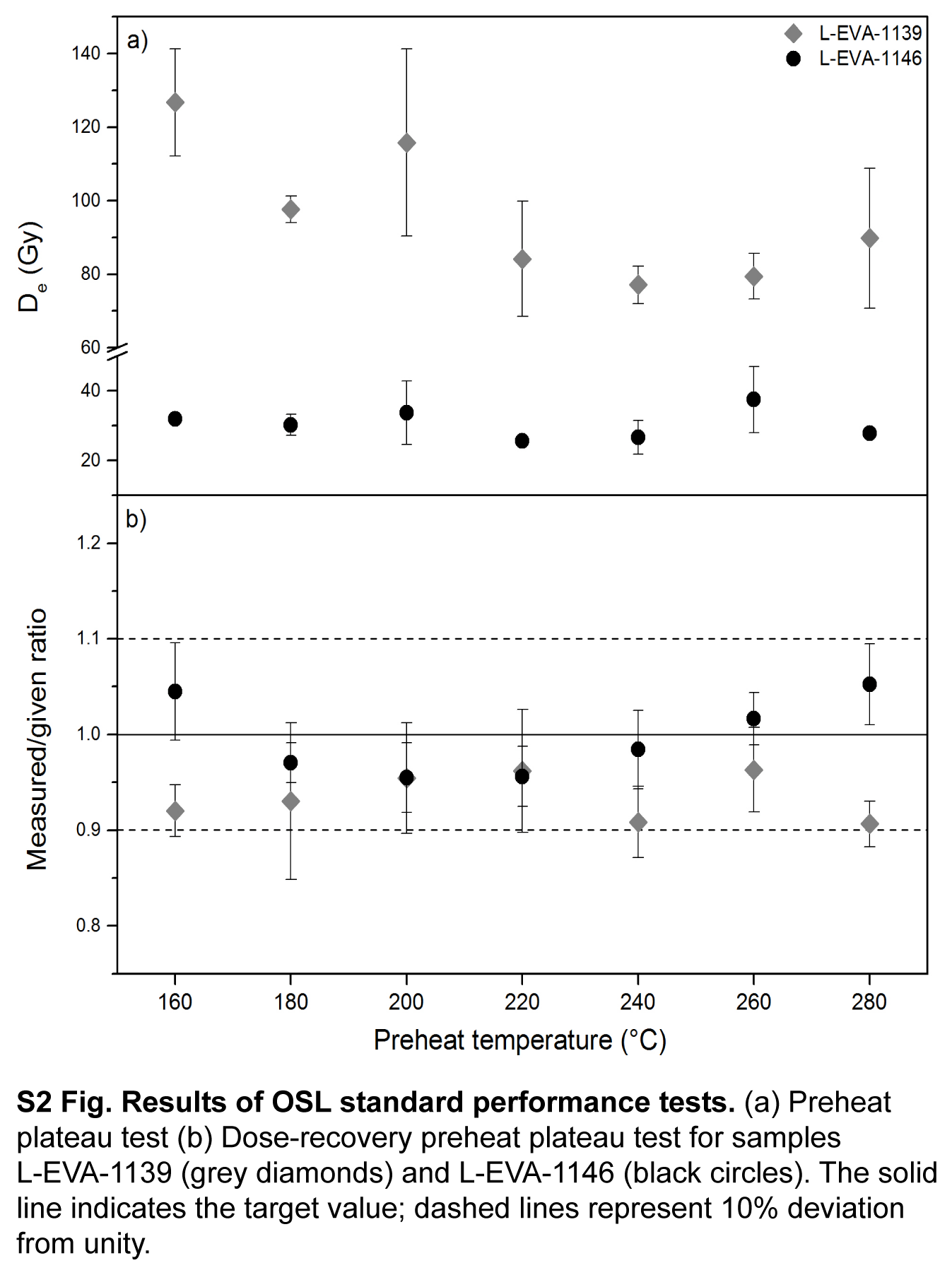

Supplement: S2 Fig — (a) Preheat plateau test (b) Dose-recovery preheat plateau test for samples L-EVA-1139 (grey diamonds) and L-EVA-1146 (black circles) are shown. The solid line indicates the target value; dashed lines represent 10% deviation from unity. (TIF) [file pone.0162280.s002.tif]

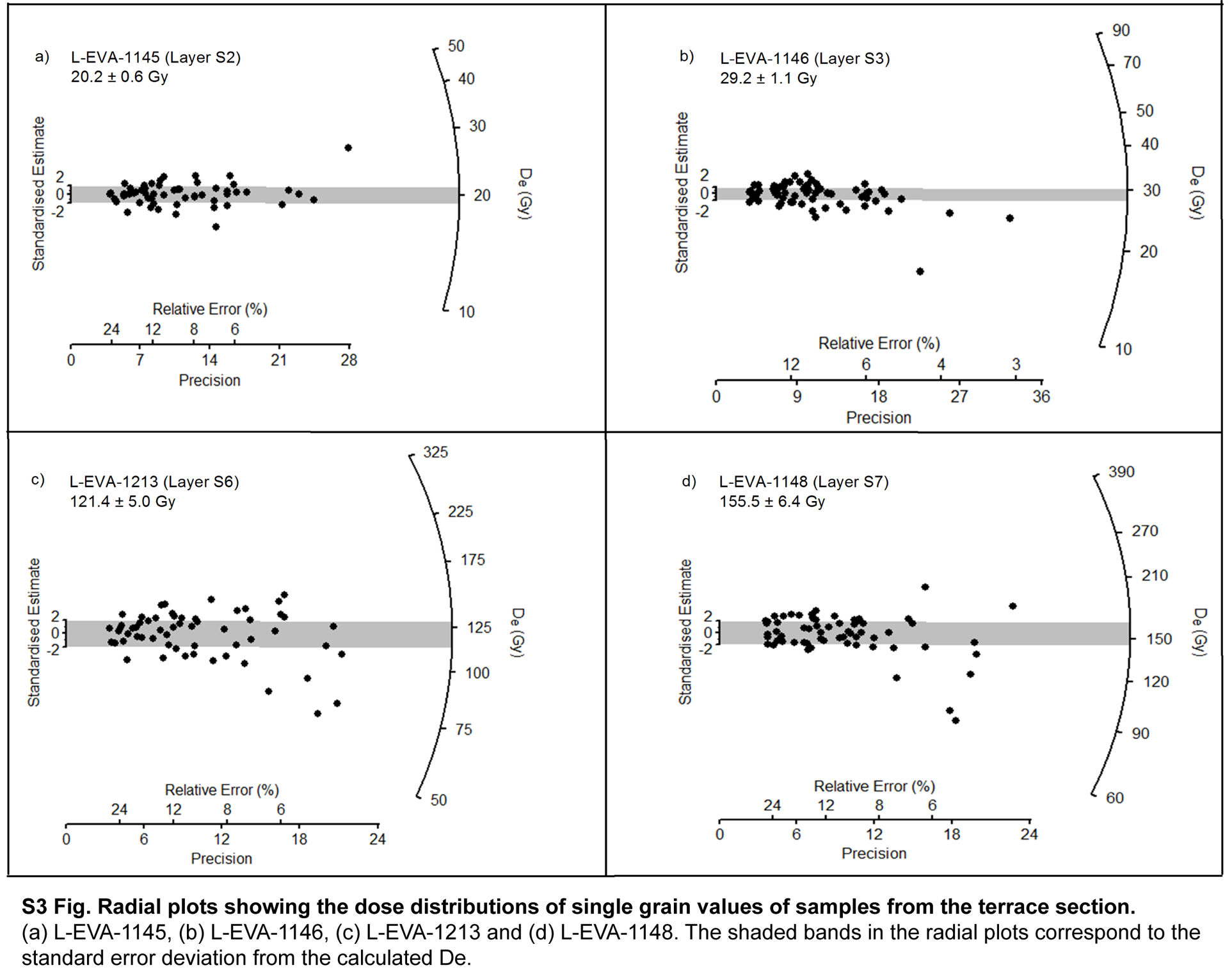

Supplement: S3 Fig — (a) L-EVA-1145, (b) L-EVA-1146, (c) L-EVA-1213 and (d) L-EVA-1148. The shaded bands in the radial plots correspond to the standard error deviation from the calculated De. (TIF) [file pone.0162280.s003.tif]

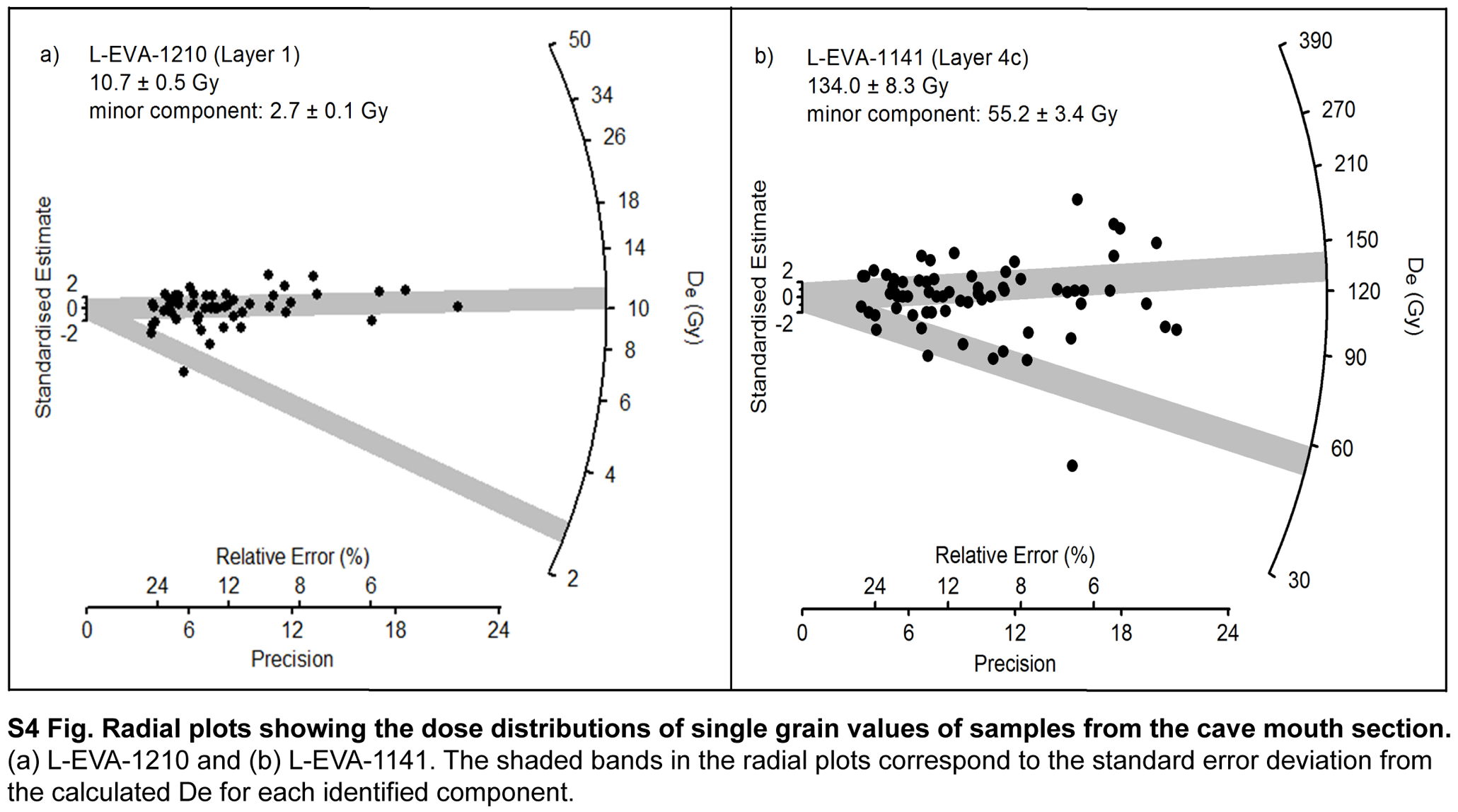

Supplement: S4 Fig — (a) L-EVA-1210 and (b) L-EVA-1141. The shaded bands in the radial plots correspond to the standard error deviation from the calculated De for each identified component. (TIF) [file pone.0162280.s004.tif]

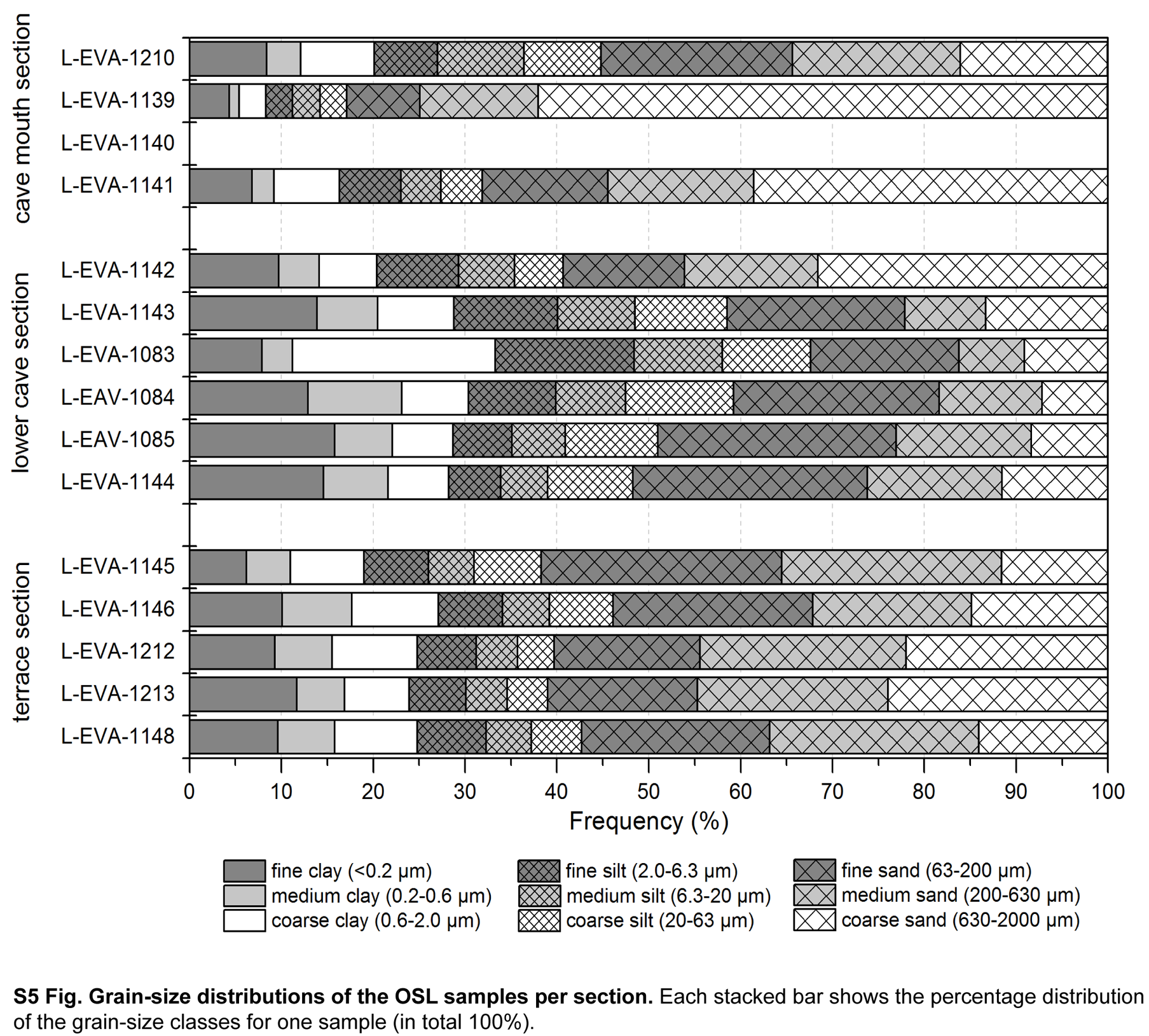

Supplement: S5 Fig — Each stacked bar shows the percentage distribution of the grain-size classes for one sample (in total 100%). (TIF) [file pone.0162280.s005.tif]

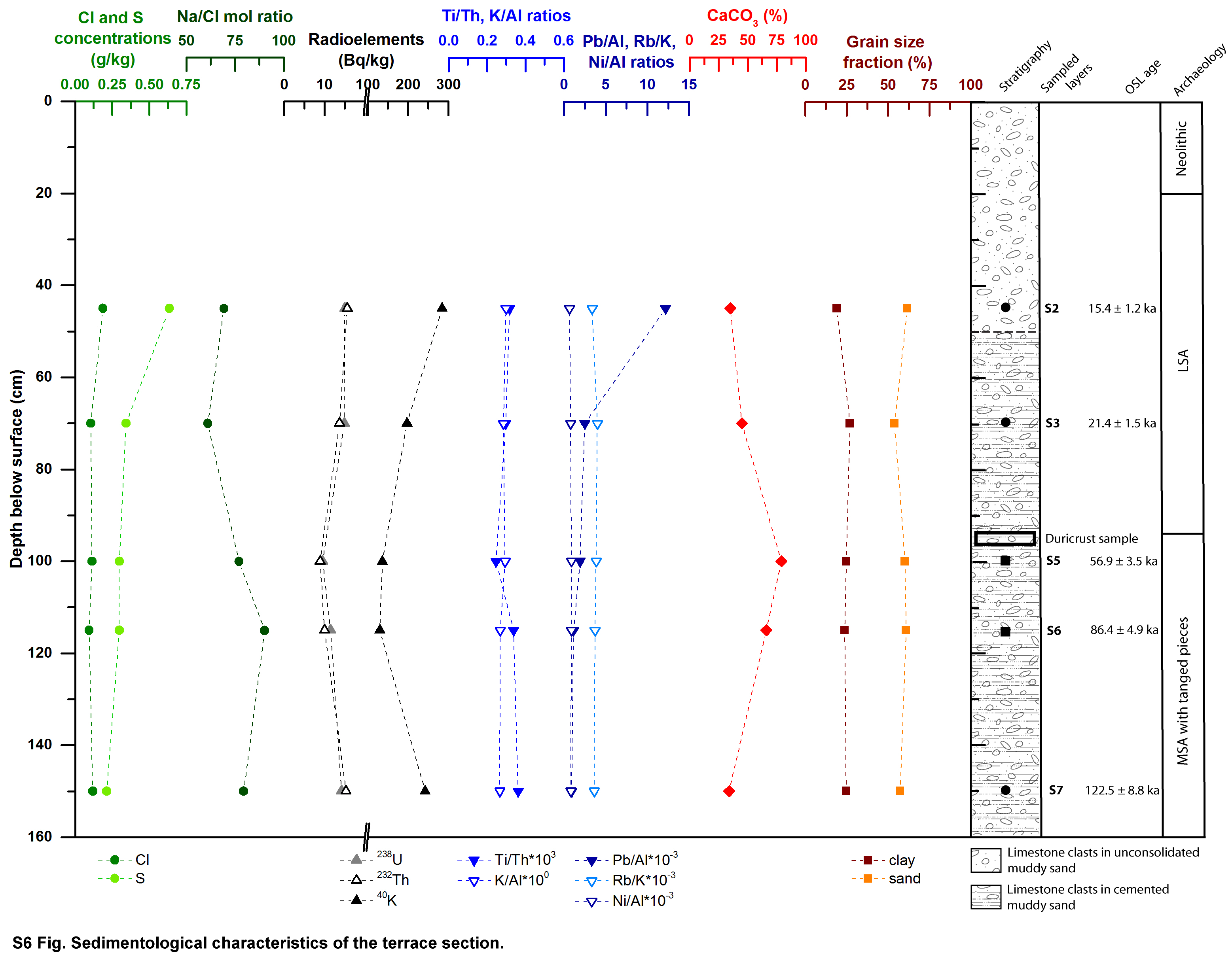

Supplement: S6 Fig — (TIF) [file pone.0162280.s006.tif]

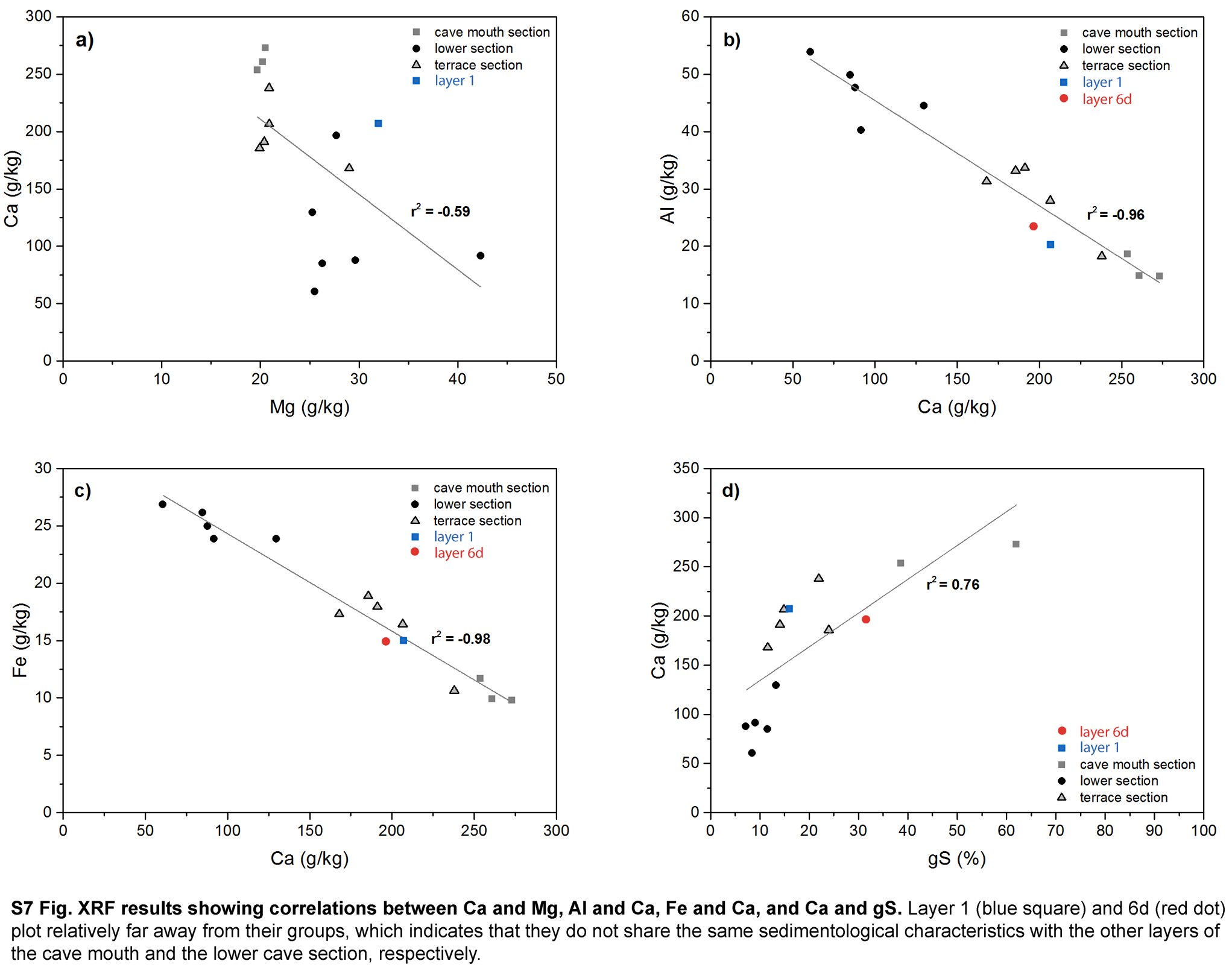

Supplement: S7 Fig — Layer 1 (blue square) and 6d (red dot) plot relatively far away from their groups, which indicates that they do not share the same sedimentological characteristics with the other layers of the cave mouth and the lower cave section, respectively. (TIF) [file pone.0162280.s007.tif]

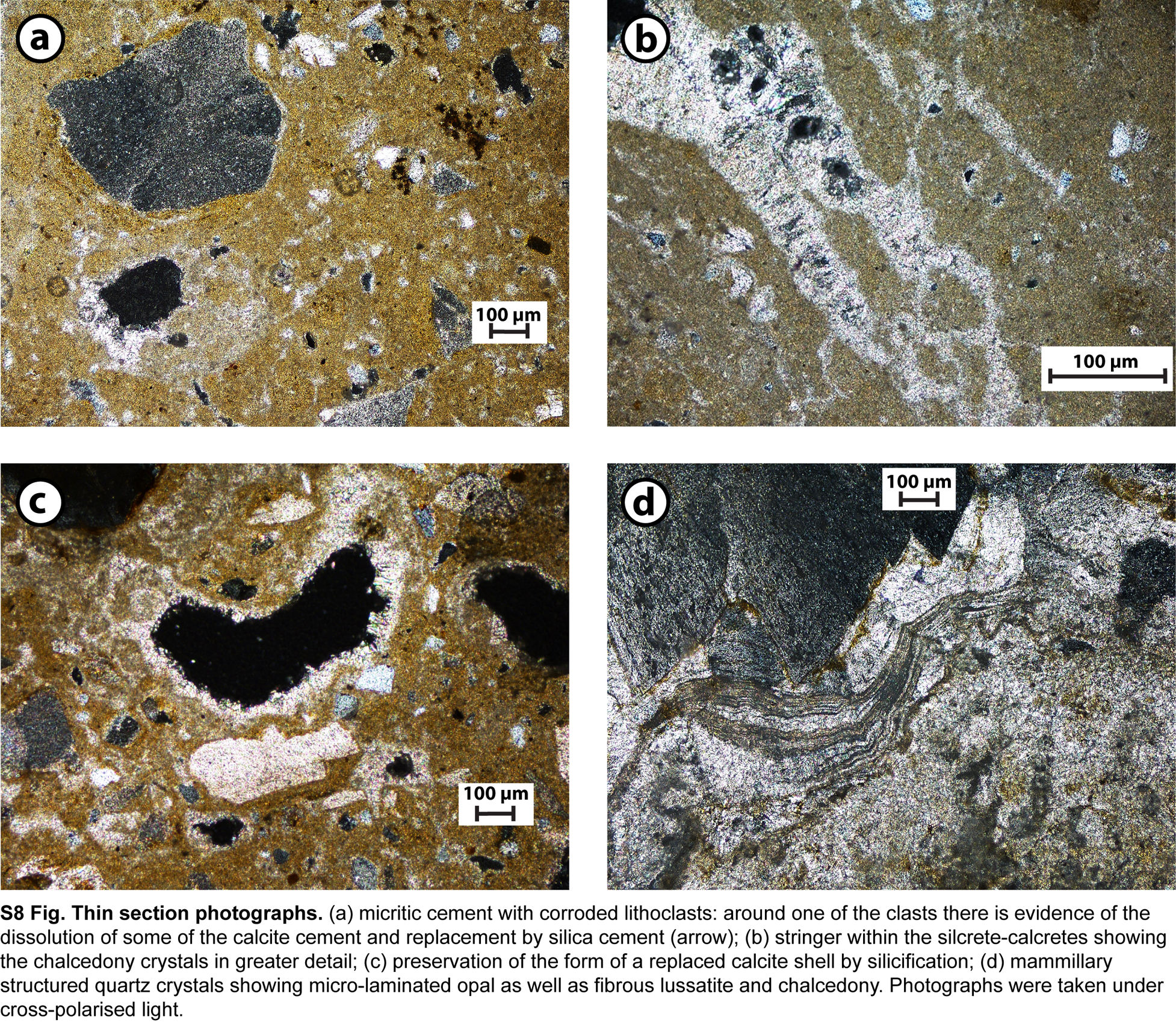

Supplement: S8 Fig — (a) micritic cement with corroded lithoclasts: around one of the clasts there is evidence of the dissolution of some of the calcite cement and replacement by silica cement (arrow); (b) stringer within the silcrete-calcretes showing the chalcedony crystals in greater detail; (c) preservation of the form of a replaced calcite shell by silicification; (d) mammillary structured quartz crystals showing micro-laminated opal as well as fibrous lussatite and chalcedony. Photographs were taken under cross-polarised light. (TIF) [file pone.0162280.s008.tif]

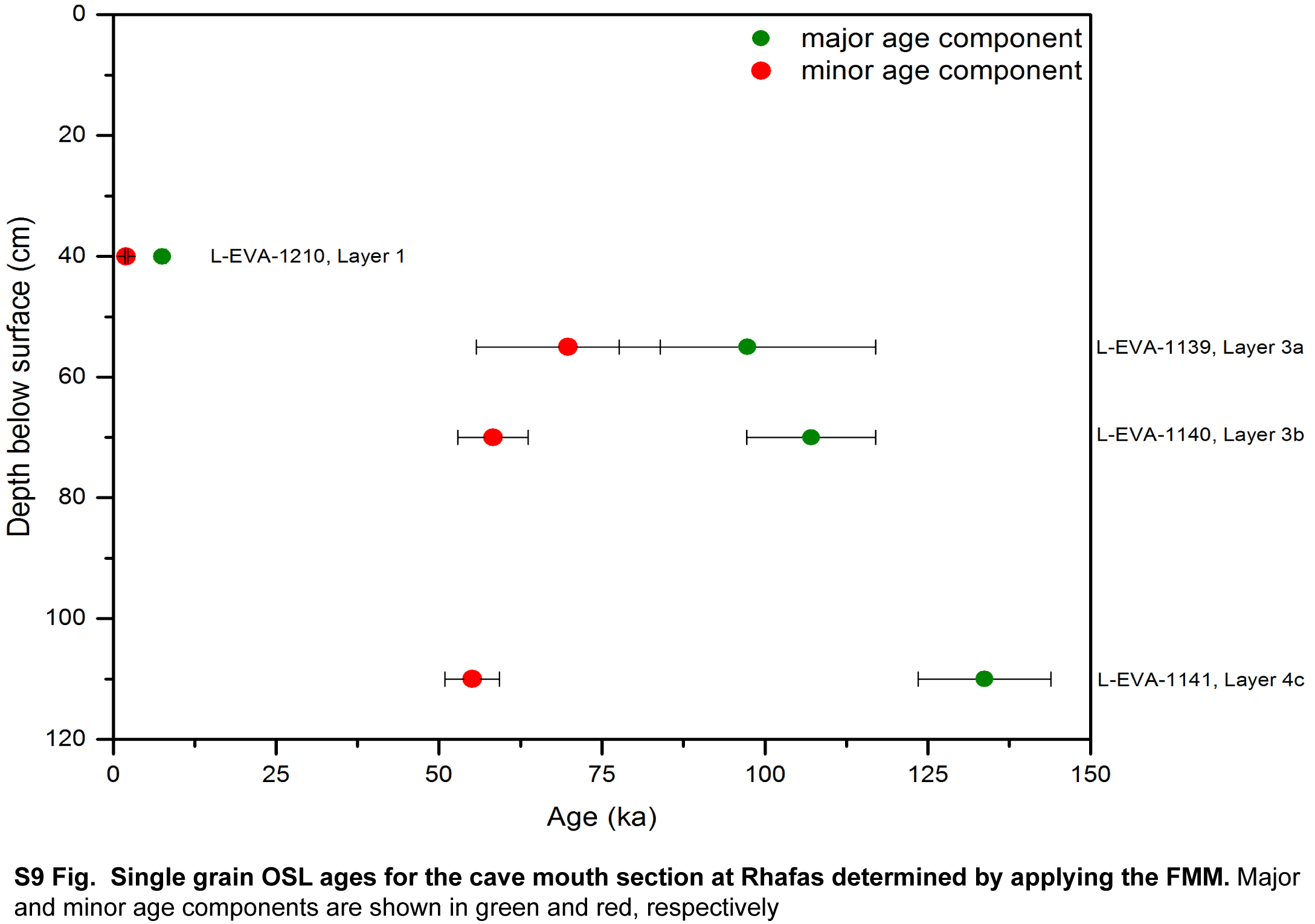

Supplement: S9 Fig — Major and minor age components are shown in green and red, respectively (TIF) [file pone.0162280.s009.tif]

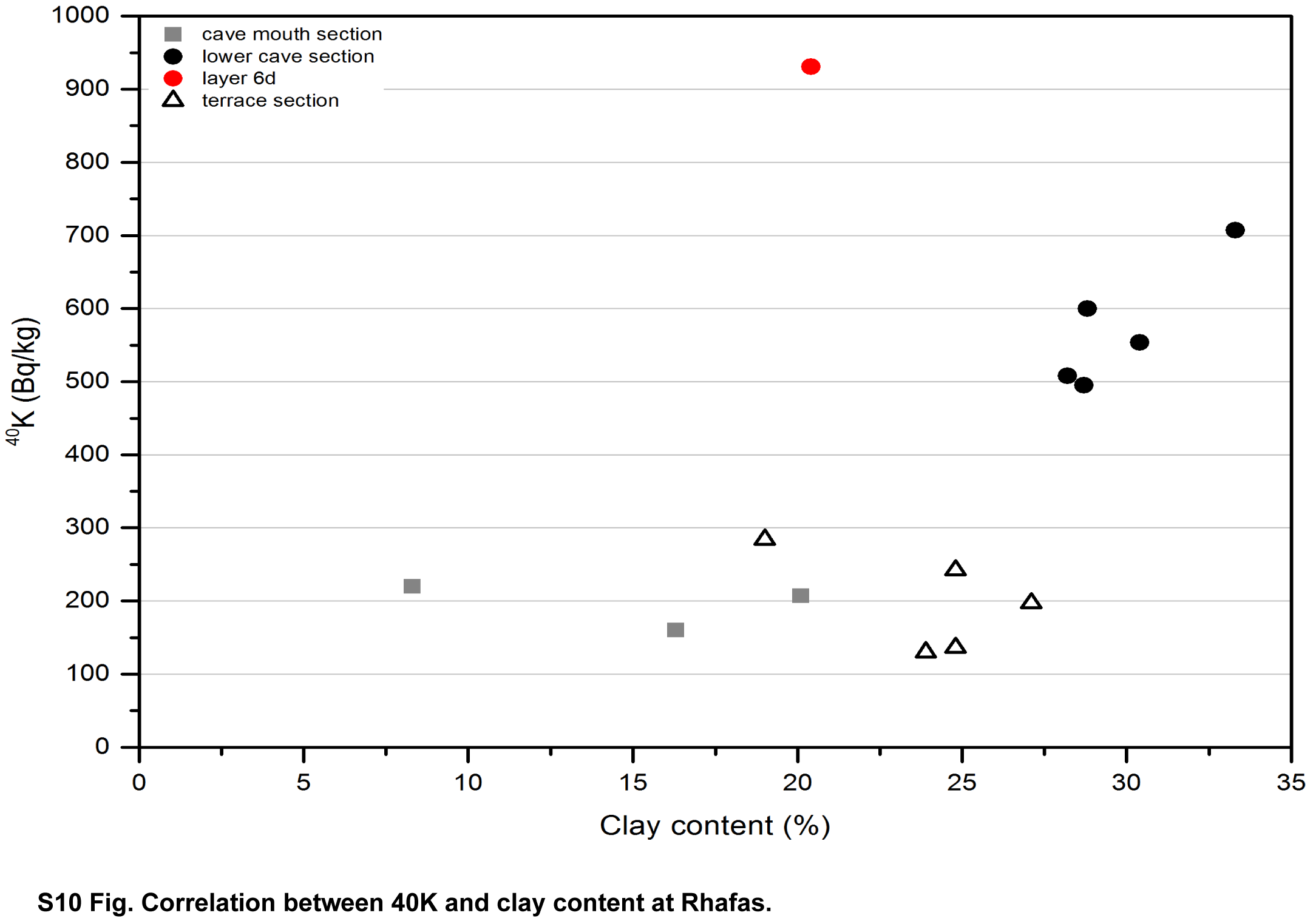

Supplement: S10 Fig — (TIF) [file pone.0162280.s010.tif]
