# Supplementary material for: A New Chronology for Rhafas, Northeast Morocco, Spanning the North African Middle Stone Age through to the Neolithic
Source: PLoS One. 2016 Sep 21;11(9):e0162280. doi: 10.1371/journal.pone.0162280 (PMC5031315; doi:10.1371/journal.pone.0162280)
Supplement: S1 File — (PDF) [file pone.0162280.s012.pdf]

S1 File

MSA/MP nomenclature in the Maghreb.

The Maghreb (comprising Morocco, Algeria, Tunisia and western Libya) is geographically located in the transition zone between the Middle/Upper Paleolithic (MP/UP) industries of western Eurasia and the African MSA/LSA [1]. The geographical position and characteristics of stone tool morphologies of the Maghreb inevitably raise the question regarding nomenclature for the Palaeolithic technocomplexes in the region. Although recent papers invest much effort into resolving the ongoing scientific debate by providing critical reviews on the characteristics of these stone tool assemblages [2, 3], an overall accepted terminology for the Palaeolithic industries from the Maghreb has yet to be agreed upon. Dibble et al. [2] pointed out that the attribution of many assemblages from Morocco to the MP seem to have happened largely due to historical reasons, and that they moreover share closer affinity to other MSA industries from the African continent. For our paper, we decided to use the African terminology and consequently use the terms MSA and LSA for the respective Palaeolithic industries from the Maghreb.

References:

1. Garcea EAA. The Spread of Aterian Peoples in North Africa. In: Garcea EAA, editor. South-Eastern Mediterranean Peoples Between 130,000 and 10,000 Years Ago. Oxford: Oxbow Books; 2010. p. 37-53.
2. Dibble HL, Aldeias V, Jacobs Z, Olszewski DI, Rezek Z, Lin SC, et al. On the industrial attributions of the Aterian and Mousterian of the Maghreb. Journal of Human Evolution. 2013;64(3):194-210. doi: http://dx.doi.org/10.1016/j.jhevol.2012.10.010.
3. Linstädter J, Eiwanger J, Mikdad A, Weniger G-C. Human occupation of Northwest Africa: A review of Middle Palaeolithic to Epipalaeolithic sites in Morocco. Quaternary International. 2012;274:158-74.
