# Supplementary material for: A New Chronology for Rhafas, Northeast Morocco, Spanning the North African Middle Stone Age through to the Neolithic
Source: PLoS One. 2016 Sep 21;11(9):e0162280. doi: 10.1371/journal.pone.0162280 (PMC5031315; doi:10.1371/journal.pone.0162280)
Supplement: S2 File — (PDF) [file pone.0162280.s013.pdf]

Sediment and bedrock analyses

Grain size

Subsamples of 10 g (<2 mm diameter) were treated with hydrogen peroxide (50 ml, 35%) overnight and heated during the next day to remove any organic matter. Afterwards, the samples were dispersed using sodium pyrophosphate solution (10 ml, 0.4 N) and ultrasonic treatment for 45 minutes. Carbonates were not dissolved, in order to retain the original grain sizes of each sample. Particle-size analysis of the sand fraction was carried out by dry-sieving with screens of 63, 125, 200 and 630 µm. The subfractions of silt and clay were determined by X-ray granulometry using a SediGraph III^{TM} with MasterTech 052 Autosampler^{TM} (Micrometrics) [1].

XRF analysis

XRF analyses were applied to sediment samples and bedrock materials to obtain their elemental compositions. Concentrations of specific elements and their ratios can serve as indicators for sediment provenience and weathering processes.

Bedrock materials were put in a steel cylinder, closed with a steel pin and crushed manually by hammering on the steel pin. The crushed bedrock samples and subsamples of the air-dried sediments (8 g, <2 mm diameter) were homogenised and milled to fine powder (<30 µm) using a vibration mill MM 200 (Retsch) for 10 min at 30 Hz). 4 g of the milled powder was mixed and homogenised with a binder (1 g Cereox Licowax). Subsequently, the mixture was filled into a die and pressed to pellets by a Vaneox press (20 t for 2 min). Analyses were carried out with a Spectro Xepos X-ray fluorescence analyser under a He gas atmosphere. Contents of the elements from sodium (11) to uranium (92) were simultaneously determined.

Thin sections

For the preparation of thin sections, samples were first impregnated with araldite glue and then placed in a vacuum (-20 in Hg) overnight. After being oven dried, both the samples and the glass slides were ground down to create flat surfaces using a Metaserv 2000 grinder. The samples were mounted onto the slides before being cut and then ground down using the diamond wheel on a PetroThin machine until a uniform thickness of 30 µm was achieved. Visual examinations were performed under a Nikon polarizing microscope using both plane-polarized and cross-polarized light (see the sections on micromorphological characteristics in Nash and McLaren [2] and the references therein).

Stable isotopes

For stable isotope analysis hand specimens were ground in a mortar using a pestle and then powdered in a ball mixer mill. About 1.5 mg per sample were flushed with He to remove air and then acidified with ~0.2 ml phosphoric acid (100%). CO_2 gas from the acidified samples was obtained using a Gilson autosampler system. Measurements were conducted using a SERCON Hydra 20-20 continuous flow isotope ratio mass spectrometer, calibrated via IAEA calcite standard CO8. The precision of carbonate analysis based on replicate investigation of the laboratory standard is better than 0.2‰ for both $\delta^{18}O$ and $\delta^{13}C$. Isotope values are reported per mil (‰) vs V-PDB.

References:

1. Zielhofer C, Clare L, Rollefson G, Wächter S, Hoffmeister D, Bareth G, et al. The decline of the early Neolithic population center of 'Ain Ghazal and corresponding earth-surface processes, Jordan Rift Valley. Quaternary Research. 2012;78(3):427-41. doi: http://dx.doi.org/10.1016/j.yqres.2012.08.006.
2. Nash DJ, McLaren SJ, editors. Geochemical Sediments and Landscapes. Oxford: Blackwell; 2007.
