# Supplementary material for: A New Chronology for Rhafas, Northeast Morocco, Spanning the North African Middle Stone Age through to the Neolithic
Source: PLoS One. 2016 Sep 21;11(9):e0162280. doi: 10.1371/journal.pone.0162280 (PMC5031315; doi:10.1371/journal.pone.0162280)
Supplement: S3 File — (PDF) [file pone.0162280.s014.pdf]

Duricrust characteristics

Thin section microscopy identified the carbonates immediately overlying Layer S5 (S6 Fig) as a diagenetically complex and well indurated duricrust, ranging in composition from calcrete to intergrade duricrusts through to silcrete. Duricrusts are geochemical sediments that form a zone of accumulation of soluble chemical precipitates within or replace underlying deposits through the movement of mineral-bearing waters [1]. Intergrade duricrusts contain a mix of silica and calcium carbonate and by comparison are generally unstable over the short to medium term, often undergoing modification, replacement and recrystallisation [2].

The smallest component of the duricrust layer comprises a calcrete *sensu stricto*. It is well indurated and massive (Fig 4a), with very little porosity remaining. Where clasts are present they are floating in the micritic matrix and the quartz grains often show evidence of corrosion at their edges. Rhizoliths (preservation of roots in mineral matter) are rare; patches of dark organic matter are more common. Irregularly coating the cement is a secondary iron (haematite) staining that is most likely derived from the local bedrock. Present at various levels with multiple cross-cutting orientations are mineral-filled stringers that extend over centimetres and are most likely related to roots [e.g. 3, 4]. There is evidence of a thin laminar crust which often develops uppermost in pedogenic calcretes [5]. Analyses on the isotopic composition were undertaken on subsamples from the calcrete itself, as well as from organic layers and the laminar crust (S3 Table). Mean values for $\delta^{13}C$ are -6.14, -2.03 and -9.12 and for $\delta^{18}O$ -6.39, -8.82 and -5.33 for the calcrete, the organic layer and the laminar crust, respectively.

Two types of intergrade duricrust exist: silcrete-calcrete and calcrete-silcrete. Silcrete development is commonly associated with the replacement of varying degrees of calcrete [6], and often shows evidence of overprinting of the calcrete fabric [7]. In the sample the silcrete-calcretes comprise predominantly

massive carbonate micrite with quartz clasts that are frequently fretted around their edges and replaced by calcrete. Although calcite cement dominates over silicate, there is the gradual destruction and loss of calcrete and replacement with silica cement representing an early stage silicification process (S8a Fig). Another feature is associated with root-related stringers that trend roughly in the same direction (Fig 4b; S8b Fig) and often contain an infilling of quartz and chalcedony [8, 9]. Calcrete-silcrete intergrade duricrusts are less common in the sample. They are dominated by silica cement over calcrete as a result of increasing silicification (Fig 4c).

A silcrete matrix may consist of a number of different polymorphs of silica, including opal, chalcedony and quartz. These stages of silicification mature over time in order of increasing crystal organisation [10]. The earliest phases of silica cements tend to be amorphous and isotropic opal, followed by lussatite (Fig 4d). The next phase of silicification is frequently chalcedony and finally quartz micro- and mega- forms [11, 12]. In the sample, mammillary-structured crystals of micro-laminated brown opal, chalcedony and lussatite are present in some voids (Fig 4d; S8d Fig). It has been argued that lussatite, chalcedony and quartz crystallise out from mobile silica in solution, whereas brown opal has formed as a result of changes to clays and haematite in the rock after it has formed [13, 14].

References:

1. McLaren S, Nash DJ. Geochemical Sediments and Landscapes: general summary. In: Nash DJ, McLaren S, editors. Geochemical Sediments and Landscapes. Oxford: Blackwell; 2007. p. 443-7.
2. Bustillo MA. Chapter 3 Silicification of Continental Carbonates. In: Alonso-Zarza AM, Tanner LH, editors. Developments in Sedimentology. Volume 62: Elsevier; 2010. p. 153-78.
3. Wright VP. Paleosols in shallow marine carbonate sequences. Earth-Science Reviews. 1994;35(4):367-95. doi: http://dx.doi.org/10.1016/0012-8252(94)90002-7.
4. Wright VP. Calcretes. In: Nash DJ, McLaren SJ, editors. Geochemical Sediments and Landscapes. Oxford: Blackwell; 2007. p. 10-45.
5. Wright VP, Platt NH, Marriott SB, Beck VH. A classification of rhizogenic (root-formed) calcretes, with examples from the Upper Jurassic-Lower Cretaceous of Spain and Upper Cretaceous of southern France — reply. Sedimentary Geology. 1997;110(3–4):305-7. doi: http://dx.doi.org/10.1016/S0037-0738(96)00091-7.
6. Thiry M, Ribet I. Groundwater silicification in Paris Basin limestones: fabrics, mechanisms, and modeling. Journal of Sedimentary Research. 1999;69:171-83.

7. Ringrose S, Harris C, Huntsman-Mapila P, Vink BW, Diskins S, Vanderpost C, et al. Origins of strandline duricrusts around the Makgadikgadi Pans (Botswana Kalahari) as deduced from their chemical and isotope composition. Sedimentary Geology. 2009;219(1–4):262-79. doi: http://dx.doi.org/10.1016/j.sedgeo.2009.05.021.
8. Summerfield MA. Silcrete. In: Goudie AS, K. P, editors. Chemical Sediments and Geomorphology. London: Academic Press; 1983. p. 55-91.
9. Summerfield MA. Petrography and diagenesis of silcrete from the Kalahari Basin and Cape Coastal Zone, southern Africa. Journal of Sedimentary Petrology. 1983;53:895-909.
10. Dixon J, McLaren S. Duricrusts. In: Parsons A, Abrahams A, editors. Geomorphology of Desert Environments: Springer Netherlands; 2009. p. 123-51.
11. Thiry M. The Phanerozoic Record of Lacustrine Basins and Their Environmental Geochemical evolution and paleoenvironments of the eocene continental deposits in the Paris Basin. Palaeogeography, Palaeoclimatology, Palaeoecology. 1989;70(1):153-63. doi: http://dx.doi.org/10.1016/0031-0182(89)90086-2.
12. Thiry M. Diversity of continental silicification features: examples from the Cenozoic deposits in the Paris Basin and neighbouring basements. In: Thiry M, Simon-Coinçon R, editors. Palaeoweathering, Palaeosurfaces and Related Continental Deposits. International Association of Sedimentologists, Special Publication No 27. Oxford: Blackwell Science; 1999. p. 87-127.
13. Thiry M, Milnes AR. Pedogenic and groundwater silcretes at Stuart Creek opal field, South Australia. Journal of Sedimentary Petrology. 1991;61:111-27.
14. Milnes AR, Thiry M. Chapter 14 - Silcretes. In: Martini IP, Chesworth W, editors. Developments in Earth Surface Processes. Volume 2: Elsevier; 1992. p. 349-77.
