# Supplementary material for: A New Chronology for Rhafas, Northeast Morocco, Spanning the North African Middle Stone Age through to the Neolithic
Source: PLoS One. 2016 Sep 21;11(9):e0162280. doi: 10.1371/journal.pone.0162280 (PMC5031315; doi:10.1371/journal.pone.0162280)
Supplement: S1 Table — (PDF) [file pone.0162280.s015.pdf]

S1 Table

Measured moisture contents, beta dose rates and chosen preheat/cutheat temperatures.

Sample	Unit	Depth (cm)	Moisture content (%)		Attenuated beta dose rate (Gy/ka)		Preheat/Cutheat
			Full saturation	Present day	Beta counter	HRGS[b]	
Cave mouth section							
L-EVA-1210	1	40	19.8	5.5[a]	0.90±0.02	0.70±0.02	260/220
L-EVA-1139	3a	55	22.9	0.9	0.73±0.01	0.74±0.01	260/220
L-EVA-1140	3b	70	22.4	1.1	0.69±0.03	0.40±0.02	260/220
L-EVA-1141	4c	110	19.2	2.2	0.68±0.01	0.56±0.01	240/200
Lower cave section							
L-EVA-1142	6d	185	24.3	1.9	1.49±0.02	2.55±0.05	260/220
L-EVA-1143	16	210	24.3	5.5	1.74±0.05	1.71±0.03	260/220
L-EVA-1083	30	260	26.8	9.3	2.32±0.05	1.99±0.04	260/220
L-EVA-1084	39	300	25.2	9.5	1.87±0.03	1.71±0.04	260/220
L-EVA-1085	55	375	26.7	5.2	1.81±0.03	1.50±0.03	260/220
L-EVA-1144	55	375	26.7	5.5	1.72±0.04	1.58±0.03	260/220
Terrace section							
L-EVA-1145	S2	45	25.0	12.1[a]	0.76±0.02	0.89±0.02	260/220
L-EVA-1146	S3	70	24.5	4.1[a]	0.79±0.01	0.73±0.02	260/220
L-EVA-1212	S5	100	19.4	4.1[a]	0.62±0.01	0.49±0.01	260/220
L-EVA-1213	S6	115	17.0	3.6[a]	0.93±0.02	0.51±0.02	260/220
L-EVA-1148	S7	150	23.8	8.1[a]	0.81±0.02	0.84±0.02	260/220

[a]Samples were collected after a rain event.

[b]High resolution gamma spectrometry.
