# Supplementary material for: A New Chronology for Rhafas, Northeast Morocco, Spanning the North African Middle Stone Age through to the Neolithic
Source: PLoS One. 2016 Sep 21;11(9):e0162280. doi: 10.1371/journal.pone.0162280 (PMC5031315; doi:10.1371/journal.pone.0162280)
Supplement: S2 Table — (PDF) [file pone.0162280.s016.pdf]

S2 Table

Summary of faunal remains.

Linnaean Names	Vernacular Names	MIS 1	MIS 2		MIS 3	MIS 5					MIS 6		Wengler's
		1[a]	S2[a]	S3[a]-S4[b]	S5[a]	3a[a]	S6[a]	3b[a]	S7[a]	4ab[b]	4c[a]	5[b]	Unit III[b]
Hystrix cristata	crested porcupine	2											
Lepus sp.	hare	1		1									
Felis cf. *caracal*	cf. caracal	1											
Canis sp.	dog or jackal	1											1
Equus sp.	indet. equid	13	35	5	1	1		2	10	2	3	4	24
Rhinocerotidae gen. et sp. indet.	rhinoceros	1						1			1		2
Suidae gen. et sp. indet.	pig(s)	3											
Sus sp.	pig	3											
Phacochoerus africanus	warthog						1						
Gazella sp.	gazelle	5	6	1							1		1
Caprini	sheep/goat	6											
Ammotragus lervia	Barbary sheep/aoudad		1										
Alcelaphini gen. et sp. indet.	hartebeest/wildebeest	19	21										14
Bovini gen. et sp. indet.	bovine	1	4						1				1
	Small bovid(s)	35	23		2	2							4
	Small-medium bovid(s)	92	55	2	1		1		1		1	1	10
	Large-medium bovid(s)	38	66	4		2					2	3	38
	Large bovid(s)	9	11				1	2			2		16
NISP		230	222	13	4	5	3	5	12	2	10	8	115
	Coprolite											2	4
Aves	Bird												1
Struthio camelus	Ostrich eggshell	18	6							1			3
	Terrestrial tortoise	18	4		1		1						1
	Terrestrial mollusk	122	8	2	4				2				

[a]Layer dated in this study.

[b]Chronological position is undetermined.
