# Supplementary material for: A New Chronology for Rhafas, Northeast Morocco, Spanning the North African Middle Stone Age through to the Neolithic
Source: PLoS One. 2016 Sep 21;11(9):e0162280. doi: 10.1371/journal.pone.0162280 (PMC5031315; doi:10.1371/journal.pone.0162280)
Supplement: S3 Table — (PDF) [file pone.0162280.s017.pdf]

**S3 Table**

Single grain characteristics.

| Sample | $n^a$ | No signal[b] | $T_N$ signal <3x BG | No $L_N/T_N$ intersection | Dim grains[c] | Recuperation >5% | Poor recycling ratio (>20%) | Depletion by IR | $D_e$ error >30% | Grubbs test[d] | accepted grains | |
|---|---|---|---|---|---|---|---|---|---|---|---|---|
| | (100%) | (%) | (%) | (%) | (%) | (%) | (%) | (%) | (%) | (%) | total | (%) |
| Cave mouth section | | | | | | | | | | | | |
| L-EVA-1210 | 4100 | 76.2 | 0.2 | 3.2 | 12.1 | 0.1 | 3.1 | 3.4 | 0.2 | 0.1 | 54 | 1.3 |
| L-EVA-1139 | 1000 | 47.9 | 0 | 7.1 | 14.6 | 0.4 | 9.0 | 11.7 | 2.4 | 0.2 | 67 | 6.7 |
| L-EVA-1140 | 900 | 54.0 | 0 | 5.8 | 14.4 | 0.2 | 7.3 | 9.4 | 1.7 | 0.2 | 62 | 6.9 |
| L-EVA-1141 | 1000 | 52.5 | 0.2 | 17.9 | 4.3 | 2.2 | 5.7 | 10.1 | 0.5 | 0 | 66 | 6.6 |
| Terrace section | | | | | | | | | | | | |
| L-EVA-1145 | 1200 | 57.9 | 0.2 | 1.3 | 16.9 | 0.4 | 8.5 | 9.8 | 0.1 | 0.1 | 58 | 4.8 |
| L-EVA-1146 | 1300 | 69.1 | 0.2 | 1.9 | 11.5 | 0.6 | 4.9 | 6.5 | 0.2 | 0 | 68 | 5.2 |
| L-EVA-1212 | 1400 | 66.3 | 0 | 2.4 | 9.9 | 0.2 | 6.1 | 9.6 | 0.8 | 0 | 64 | 4.6 |
| L-EVA-1213 | 1500 | 67.3 | 0.3 | 7.9 | 7.3 | 0.4 | 4.5 | 7.2 | 1.1 | 0 | 59 | 3.9 |
| L-EVA-1148 | 800 | 33.9 | 0.1 | 19.3 | 13.0 | 1.9 | 7.8 | 13.5 | 2.8 | 0 | 63 | 7.9 |

[a]Total number of grains measured per sample.

[b]Percentage of grains not emitting any detectable luminescence signal.

[c]Percentage of grains rejected due to insufficient test-dose signal.

[d]Percentage of grains identified as statistical outliers [1].

1. Grubbs FE. Sample Criteria for Testing Outlying Observations. 1950:27-58. doi: 10.1214/aoms/1177729885.
