# Supplementary material for: A New Chronology for Rhafas, Northeast Morocco, Spanning the North African Middle Stone Age through to the Neolithic
Source: PLoS One. 2016 Sep 21;11(9):e0162280. doi: 10.1371/journal.pone.0162280 (PMC5031315; doi:10.1371/journal.pone.0162280)
Supplement: S4 Table — (PDF) [file pone.0162280.s018.pdf]

S4 Table

Single grain dose recovery properties.

Sample	Unit	accepted/measured grains	given dose (Gy)	measured D_e[a] (Gy)	Overdispersion (%)	recovery ratio
Cave mouth section						
L-EVA-1210	1	51/1600	11.2	10.9	2±4	0.97±0.04
L-EVA-1139	3a	86/1000	78.5	72.2	7±1	0.92±0.05
L-EVA-1140	3b	58/900	98.5	90.6	14±2	0.92±0.06
L-EVA-1141	4c	97/800	105.0	100.8	5±1	0.96±0.05
Terrace section						
L-EVA-1145	S2	88/1200	21.5	20.9	7±1	0.97±0.05
L-EVA-1146	S3	86/1400	23.9	22.9	7±1	0.96±0.05
L-EVA-1212	S5	61/900	67.2	63.8	6±1	0.95±0.05
L-EVA-1213	S6	63/900	118.3	111.2	14±2	0.94±0.07
L-EVA-1148	S7	89/800	135.2	124.4	9±1	0.92±0.05

[a]Detremined using the Central Age Model (Galbraith et al., 1999).
