# Supplementary material for: A New Chronology for Rhafas, Northeast Morocco, Spanning the North African Middle Stone Age through to the Neolithic
Source: PLoS One. 2016 Sep 21;11(9):e0162280. doi: 10.1371/journal.pone.0162280 (PMC5031315; doi:10.1371/journal.pone.0162280)
Supplement: S5 Table — (PDF) [file pone.0162280.s019.pdf]

S5 Table

Grain size results.

Sample	Layer	Grain size fractions (%)												
		fT	mT	gT	fU	mU	gU	fS	mS	gS	Total clay (T)	Total silt (U)	Total sand(S)	classified as [1, 2]
Cave mouth section														
L-EVA-1210	1	8.4	3.7	8.0	6.9	9.4	8.4	20.8	18.3	16.0	20.1	24.7	55.2	muddy Sand
L-EVA-1139	3a	4.3	1.1	2.9	2.9	3	2.9	8.0	12.9	62.0	8.3	8.8	82.9	muddy Sand
L-EVA-1140	3b	-[a]	-[a]	-[a]	-[a]	-[a]	-[a]	-[a]	-[a]	-[a]	-[a]	-[a]	-[a]	-[a]
L-EVA-1141	4c	6.8	2.4	7.1	6.7	4.4	4.5	13.7	15.8	38.6	16.3	15.6	68.1	muddy Sand
Lower cave section														
L-EVA-1142	6d	9.7	4.4	6.3	8.9	6.1	5.3	13.2	14.5	31.6	20.4	20.3	59.3	muddy Sand
L-EVA-1143	16	13.9	6.6	8.3	11.3	8.4	10.0	19.4	8.8	13.3	28.8	29.7	41.5	sandy Mud
L-EVA-1083	30	7.9	3.3	22.1	15.1	9.6	9.6	16.2	7.1	9.1	33.3	34.3	32.4	sandy Mud
L-EVA-1084	39	12.9	10.2	7.3	9.5	7.6	11.7	22.4	11.2	7.2	30.4	28.8	40.8	sandy Mud
L-EVA-1085	55	15.8	6.3	6.6	6.4	5.8	10.1	25.9	14.7	8.4	28.7	22.3	49.0	sandy Mud
L-EVA-1144	55	14.6	7.0	6.6	5.7	5.1	9.3	25.5	14.7	11.6	28.2	20.1	51.7	muddy Sand
Terrace section														
L-EVA-1145	S2	6.2	4.8	8.0	7.0	5.0	7.3	26.2	23.9	11.6	19.0	19.3	61.7	muddy Sand
L-EVA-1146	S3	10.1	7.6	9.4	7.0	5.1	6.9	21.8	17.3	14.9	27.1	19.0	53.9	muddy Sand
L-EVA-1212	S5	9.3	6.2	9.3	6.4	4.5	4.0	15.9	22.5	22.0	24.8	14.9	60.3	muddy Sand
L-EVA-1213	S6	11.7	5.2	7.0	6.2	4.5	4.4	16.3	20.7	24.0	23.9	15.1	61.0	muddy Sand
L-EVA-1148	S7	9.6	6.2	9.0	7.5	4.9	5.5	20.5	22.7	14.1	24.8	17.9	57.3	muddy Sand

[a]Not enough sample material for grain size determination available.

1. Folk RL. The distinction between grain size and mineral composition in sedimentary rocks. Journal of Geology. 1954;62:344-59.
2. Folk RL. Petrology of Sedimentary Rocks. Austin, TX: Hemphill Publishing; 1980. 184 p.
