# Supplementary material for: A New Chronology for Rhafas, Northeast Morocco, Spanning the North African Middle Stone Age through to the Neolithic
Source: PLoS One. 2016 Sep 21;11(9):e0162280. doi: 10.1371/journal.pone.0162280 (PMC5031315; doi:10.1371/journal.pone.0162280)
Supplement: S6 Table — (PDF) [file pone.0162280.s020.pdf]

S6 Table

XRF results and $CaCO_3$ content.

Sample	S	Cl	Mg	Ca	Al	Fe	Na/Cl 10^0	Pb/Al	Rb/K	Ni/Al	Ti/Th	K/Al	$CaCO_3$
	(g/kg)						mol ratio	10^{-3}	10^{-3}	10^{-3}	10^3	10^0	(%)
Cave mouth section													
L-EVA-1210	0.56	0.20	32.0	207	20.3	15.0	52.4	3.36	3.15	0.97	0.27	0.42	56
L-EVA-1139	0.65	0.16	20.5	273	14.8	9.8	67.0	2.48	3.91	3.12	0.21	0.39	78
L-EVA-1140	0.55	0.23	20.2	261	14.9	9.9	41.4	2.88	3.86	2.71	0.22	0.39	89
L-EVA-1141	0.52	0.20	19.7	254	18.7	11.7	46.7	1.57	3.81	1.37	0.23	0.35	62
Lower cave section													
L-EVA-1142	2.18	4.59	27.7	197	23.5	14.9	3.3	0.96	1.54	0.93	0.30	0.93	61
L-EVA-1143	2.92	0.96	25.6	130	44.5	23.9	10.5	0.77	2.82	0.63	0.41	0.42	33
L-EVA-1083	3.21	11.3	42.3	92	40.2	23.9	1.7	1.17	2.13	0.70	0.38	0.61	26
L-EVA-1084	2.62	4.09	29.6	88	47.7	25.0	5.0	1.09	2.54	0.66	0.39	0.45	19
L-EVA-1085	0.44	1.86	25.5	61	53.9	26.9	5.8	0.82	2.43	0.55	0.44	0.41	34
L-EVA-1144	0.88	1.02	26.3	85	49.9	26.1	12.9	0.87	2.50	0.57	0.46	0.40	21
Terrace section													
L-EVA-1145	0.64	0.19	29.0	168	31.3	17.3	69.4	12.18	3.37	0.67	0.32	0.30	35
L-EVA-1146	0.34	0.11	20.9	207	28.0	16.4	61.0	2.50	4.00	0.80	0.30	0.29	45
L-EVA-1212	0.30	0.11	20.9	240	18.3	10.7	77.1	1.93	3.85	0.89	0.25	0.29	79
L-EVA-1213	0.30	0.10	19.9	186	33.2	19.0	90.3	1.14	3.73	0.89	0.34	0.27	66
L-EVA-1148	0.21	0.12	20.4	191	33.7	17.9	79.5	0.93	3.62	0.81	0.36	0.27	34
