# Supplementary material for: A New Chronology for Rhafas, Northeast Morocco, Spanning the North African Middle Stone Age through to the Neolithic
Source: PLoS One. 2016 Sep 21;11(9):e0162280. doi: 10.1371/journal.pone.0162280 (PMC5031315; doi:10.1371/journal.pone.0162280)
Supplement: S7 Table — (PDF) [file pone.0162280.s021.pdf]

S7 Table

Summary of istope data.

Sample	$\delta^{13}C$ (‰)	$\delta^{18}O$ (‰)
**Calcrete**		
2iii	-7.65	-5.03
3i	-6.67	-6.61
3iii	-6.79	-6.33
4ii	-6.56	-6.23
5i	-4.31	-6.84
5ii	-4.87	-7.31
mean	**-6.14**	**-6.39**
standard deviation	**1.16**	**0.70**
**Organic layer**		
3iii	-2.85	-8.16
4i	-1.21	-9.48
mean	***-2.03***	***-8.82***
standard deviation	***1.64***	***0.66***
**Laminar crust**		
5iii	-9.12	-5.33
