# Supplementary material for: A New Chronology for Rhafas, Northeast Morocco, Spanning the North African Middle Stone Age through to the Neolithic
Source: PLoS One. 2016 Sep 21;11(9):e0162280. doi: 10.1371/journal.pone.0162280 (PMC5031315; doi:10.1371/journal.pone.0162280)
Supplement: S8 Table — (PDF) [file pone.0162280.s022.pdf]

S8 Table

FMM details. For L-EVA-1139 and L-EVA-1140 BIC and llik continuously increase and decrease, respectively, when Overdispersion values further increase. The chosen combination of Overdispersion and number of components used for age determination of each sample are shaded in grey.

Sample	k	σ_{OD}[1]	K1		K2		K3		BIC	llik
			D_e±error	Proportion	D_e±error	Proportion	D_e±error	Proportion		
L-EVA-1210	2	15	3.2±0.1	7.1%	10.9±0.3	92.9%			62.1	-25.09
	2	20	3.0±0.1	6.3%	10.8±0.4	93.7%			54.2	-21.13
	2	25	2.7±0.1	4.8%	10.7±0.5	95.2%			52.7	-20.36
	2	30	1.8±0.5	2.4%	10.4±1.0	97.6%			53.6	-20.81
	3	15	1.6±0.1	1.9%	5.9±0.6	14.8%	11.4±0.6	83.3%	53.0	-16.47
	3	20	1.6±0.3	1.9%	5.5±0.7	10.7%	11.2±0.4	87.4%	53.6	-16.84
	3	25	1.6±0.3	1.9%	5.0±0.8	6.9%	10.9±0.4	91.2%	56.6	-18.35
	3	30	1.6±0.4	2.0%	4.8±1.1	3.6%	10.7±0.5	94.4%	60.7	-20.39
L-EVA-1139	2	15	71.3±14.1	42.2%	99.4±19.7	57.6%			23.0	-5.20
	2	20	72.3±30.0	28.3%	92.4±23.7	71.7%			24.5	-5.93
	3	15	51.3±24.3	3.5%	77.3±8.9	54.3%	103.7±9.2	42.2%	30.3	-4.64
	3	20	69.7±20.7	20.4%	89.5±NaN	45.5%	92.87±57.1	37.2%	30.9	-4.93
L-EVA-1140	2	15	57.2±3.4	25.9%	105.8±7.5	74.1%			55.9	-21.76
	2	20	56.7±4.6	23.1%	104.2±8.5	76.9%			51.4	-19.51
	2	25	57.1±13.5	19.9%	101.6±9.3	80.1%			53.0	-20.29
	3	15	53.4±7.7	19.3%	91.3±10.5	48.8%	122.9±12.5	31.9%	59.1	-19.23
	3	20	56.0±10.7	21.9%	97.0±12.2	38.1%	110.0±5.0	40.1%	59.6	-19.48
L-EVA-1141	2	15	59.1±2.4	21.6%	137.7±3.2	78.4%			133.0	-60.20
	2	20	56.7±3.0	18.6%	135.7±4.0	81.4%			106.2	-46.80
	2	25	55.2±3.4	16.4%	134.0±8.3	83.6%			96.1	-41.77
	2	30	53.6±7.3	13.7%	131.4±9.4	86.3%			99.3	-45.37
	3	15	50.8±7.5	14.3%	114.9±4.5	60.8%	191.6±7.9	24.9%	109.0	-44.03
	3	20	51.7±10.1	14.3%	118.2±6.0	65.4%	195.3±13.0	20.3%	103.0	-41.04
	3	25	51.9±13.9	13.5%	120.8±9.3	69.4%	188.1±23.1	17.1%	101.9	-40.46
	3	30	51.8±22.8	12.1%	115.6±NaN	35.1%	140.4±NaN	52.8%	101.6	-40.34

[1]Overdispersion used running the FMM.
